# An ER Complex of ODR-4 and ODR-8/Ufm1 Specific Protease 2 Promotes GPCR Maturation by a Ufm1-Independent Mechanism

**DOI:** 10.1371/journal.pgen.1004082

**Published:** 2014-03-06

**Authors:** Changchun Chen, Eisuke Itakura, Katherine P. Weber, Ramanujan S. Hegde, Mario de Bono

**Affiliations:** MRC Laboratory of Molecular Biology, Cambridge, United Kingdom; University of California San Diego, United States of America

## Abstract

Despite the importance of G-protein coupled receptors (GPCRs) their biogenesis is poorly understood. Like vertebrates, *C. elegans* uses a large family of GPCRs as chemoreceptors. A subset of these receptors, such as ODR-10, requires the *odr-4* and *odr-8* genes to be appropriately localized to sensory cilia. The *odr-4* gene encodes a conserved tail-anchored transmembrane protein; the molecular identity of *odr-8* is unknown. Here, we show that *odr-8* encodes the *C. elegans* ortholog of Ufm1-specific protease 2 (UfSP2). UfSPs are cysteine proteases identified biochemically by their ability to liberate the ubiquitin-like modifier Ufm1 from its pro-form and protein conjugates. ODR-8/UfSP2 and ODR-4 are expressed in the same set of twelve chemosensory neurons, and physically interact at the ER membrane. ODR-4 also binds ODR-10, suggesting that an ODR-4/ODR-8 complex promotes GPCR folding, maturation, or export from the ER. The physical interaction between human ODR4 and UfSP2 suggests that this complex's role in GPCR biogenesis may be evolutionarily conserved. Unexpectedly, mutant versions of ODR-8/UfSP2 lacking catalytic residues required for protease activity can rescue all *odr-8* mutant phenotypes tested. Moreover, deleting *C. elegans ufm-1* does not alter chemoreceptor traffic to cilia, either in wild type or in *odr-8* mutants. Thus, UfSP2 proteins have protease- and Ufm1-independent functions in GPCR biogenesis.

## Author Summary

Despite the importance of G-protein coupled receptors (GPCRs), we know little about their biogenesis. Olfactory receptors form a large and divergent group of GPCRs. We investigate their biogenesis in *C. elegans*. We show that maturation of a subset of these GPCRs, including the diacetyl receptor ODR-10, requires Ufm1 specific protease 2 (UfSP2), which corresponds to *odr-8*. Biochemical studies suggest mouse UfSP2 activates the Ubiquitin-like molecule Ufm1 and cleaves it from protein conjugates. However, neither the protease active site nor *ufm-1* is required for UfSP2/ODR-8 to promote ODR-10 maturation. *C. elegans* UfSP2 is expressed in the same chemosensory neurons as ODR-4, a tail-anchored transmembrane protein also required for ODR-10 maturation. ODR-4 resides in the endoplasmic reticulum (ER); UfSP2 is cytosolic but associates with ER membranes. In *odr-4* and *odr-8* mutants ODR-10-GFP is retained in the ER, suggesting these genes are required to fold GPCRs or traffic them out of the ER. ODR-4 interacts biochemically with ODR-8 and ODR-10 to form an ER complex. ODR-4 and UfSP2 are conserved from plants to man, and human ODR4 can bind human UfSP2 and recruit it to ER membranes. Both proteins are expressed widely in mammals, suggesting a broader role in GPCR biogenesis.

## Introduction

Molecular chaperones ensure the correct folding, assembly, quality control, traffic, and sub-cellular targeting of newly made proteins. Failure of these processes results in protein aggregation, with potential pathological consequences [1]. In the nervous system a growing number of chaperones have been identified that are specialized to facilitate the biogenesis of specific molecules [2]–[3]. Together with general chaperones, these molecules prevent accumulation of protein aggregates in neurons and provide protection against neurodegeneration [4].

GPCRs form a large family of polytopic transmembrane proteins that share a common fold [5]. Despite their importance, the biogenesis of GPCRs is poorly understood. GPCRs are co-translationally targeted to and inserted into the endoplasmic reticulum (ER) membrane using the canonical translocon machinery [6]. In the ER, GPCRs are often N-glycosylated and fold with the help of chaperones [7]–[9]. General chaperones implicated in GPCR folding include calnexin and calreticulin. More specific chaperones have also been identified for a few receptors, including RTP1 and RTP2 for some mammalian olfactory receptors [10], XPORT and NinaA for fly rhodopsin [11], and DRiP78 for the D1 dopamine receptor [12]. GPCR assembly in the ER is monitored by poorly defined quality control systems, and improperly folded receptors are targeted for refolding or degradation by the ubiquitin-proteasome system [9]. Molecules passing ER quality control are exported to downstream compartments of the secretory pathway. Motifs involved in GPCR ER export have been defined for some receptors, [12], although some of these may be involved in correct GPCR folding, a pre-requisite for export [13]. In the Golgi and trans-Golgi network GPCRs are further glycosylated, and assembled into vesicles appropriate for traffic to their appropriate sub-cellular localization. In neurons these destinations can include dendrites, axons or synapses. While the molecular machineries mediating many steps in GPCR biogenesis are unknown, it is clear that ER retention of GPCRs due to misfolding leads to disease, including retinitis pigmentosa [14], nephrogenic diabetes insipidus [15], and hypogonadotropic hypogonadism [16].

In the nematode *C. elegans*, like in vertebrates, GPCRs play central roles in chemoreception [17]–[19]. The *C. elegans* genome encodes more than 1300 predicted GPCR chemoreceptors of widely divergent sequence [18]. Many of these GPCRs appear to be expressed in one or more of 12 gustatory and olfactory neuron types [17]. The most intensively studied of these receptors, ODR-10, mediates *C. elegans* chemotaxis to the volatile odor diacetyl. ODR-10 is specifically expressed in the AWA olfactory neuron, and localizes to sensory cilia [19]. Screens for odortaxis defective mutants have identified two loci that disrupt ODR-10 localization to cilia, *odr-4* and *odr-8* [20]. In these mutants ODR-10-GFP is retained in endomembranes in the cell body. *odr-4* and *odr-8* are only required for localization of a subset of GPCR chemoreceptors, and do not affect localization of other signal transduction components, such as G-proteins and ion channels, to cilia [20]. These genetic data suggest that *odr-4* and *odr-8* encode accessory proteins involved in the maturation, traffic or localization of GPCRs.

As well as being required for olfactory responses, *odr-4* and *odr-8* are required for *npr-1* animals to aggregate efficiently [21] and to respond to variation in ambient oxygen [22]. Our interest in the molecular basis of aggregation behaviour motivated us to understand ODR-4 and ODR-8. Earlier work had established that *odr-4* encodes a tail-anchored transmembrane protein selectively expressed in 12 *C. elegans* chemosensory neurons and localized to unidentified intracellular membrane compartments [20]. The molecular identity of *odr-8* has remained unknown. Here, we identify the gene encoded by *odr-8* and provide a characterization of its relationship to *odr-4* and its role in GPCR biogenesis.

## Results

### Mutants defective in UFM1-specific protease 2 exhibit behavioral defects

**Figure 1:**
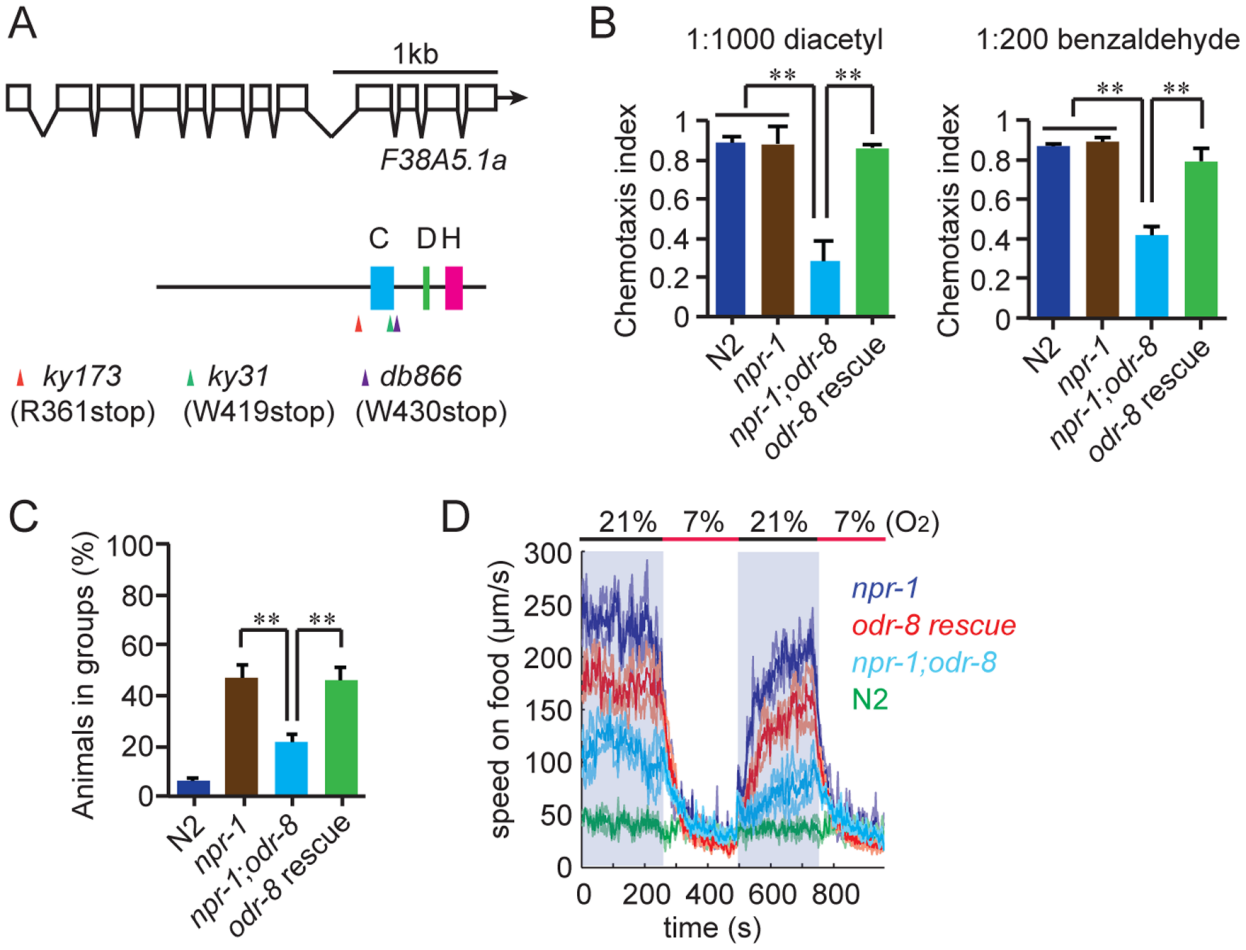
*odr-8* mutants are defective in Ufm1 specific protease 2. (A) The UfSP2 gene corresponds to ORF F38A5.1. Shown are the exon/intron structure of the gene and the location of the Cysteine (blue), Histidine (green) and Aspartate (pink) boxes containing catalytic residues in the papain fold. The location of stop codons associated with the *db866*, *ky173* and *ky31* mutations is also indicated. (B–D). A transgene carrying F38A5.1a genomic fragment rescues *odr-8* defects in olfaction (B), aggregation (C), and responses to changes in O_2_ (D). **, *p*<0.01 compared to non-transgenic controls (ANOVA, Bonferroni's multiple comparisons test).

We mutagenized *npr-1* animals, isolated mutants that failed to aggregate, and sequenced their genomes. At the same time we sequenced two strains bearing alleles of *odr-8*, *ky173* and *ky31*, which we previously showed disrupt aggregation behavior [21]. Genome sequence analysis identified only one open reading frame that had lesions in *ky31* and *ky173* alleles and mapped to the interval where *odr-8* maps genetically [20]. Three other aggregation-defective strains in our sequenced collection also had mutations in this gene, which is called F38A5.1. Of the five mutations in F38A5.1, three introduced stop codons (Figure 1A).

To establish if defects in F38A5.1 caused *odr-8* phenotypes we carried out transgenic rescue experiments. Genomic DNA bearing the F38A5.1 open reading frame rescued *odr-8* defects in odortaxis, aggregation behavior, and responses to oxygen (Figure 1B–D), confirming the molecular identity of *odr-8*.

F38A5.1 encodes the *C. elegans* ortholog of Ufm1-specific protease 2 (UfSP2). Ufm1 (Ubiquitin fold modifier) is a ubiquitin-like post-translational modifier [23]. Its tertiary structure is very similar to that of ubiquitin (Ub) although its primary sequence shows no obvious sequence similarity to Ub [24]. Like Ub, Ufm1 is synthesized as an inactive precursor that is proteolytically cleaved to expose a glycine C-terminal residue. This glycine can then be conjugated to target proteins at lysine residues, although only one Ufm1 target has been identified to date, the ER protein C20orf116 [25]. *In vitro* studies show that mouse UfSP2 can both activate pro-Ufm1 and cleave it from native protein conjugates, a process termed de-ufmylation (Figure S1) [25]–[27]. Ufm1 and UfSP2 are highly conserved across eukaryotes (Figure S1B and Figure S2), but their *in vivo* roles are unknown.

### ODR-8/UfSP2 is co-expressed with ODR-4 in chemosensory neurons

To determine where ODR-8/UfSP2 is expressed, we fused DNA encoding the fluorescent protein mCherry upstream of the *odr-8* open reading frame and expressed this transgene from the *odr-8* promoter. The construct rescued all the defects observed in *odr-8* mutants, and revealed mCherry-ODR-8 expression in a small number of head neurons and two tail neurons.

**Figure 2:**
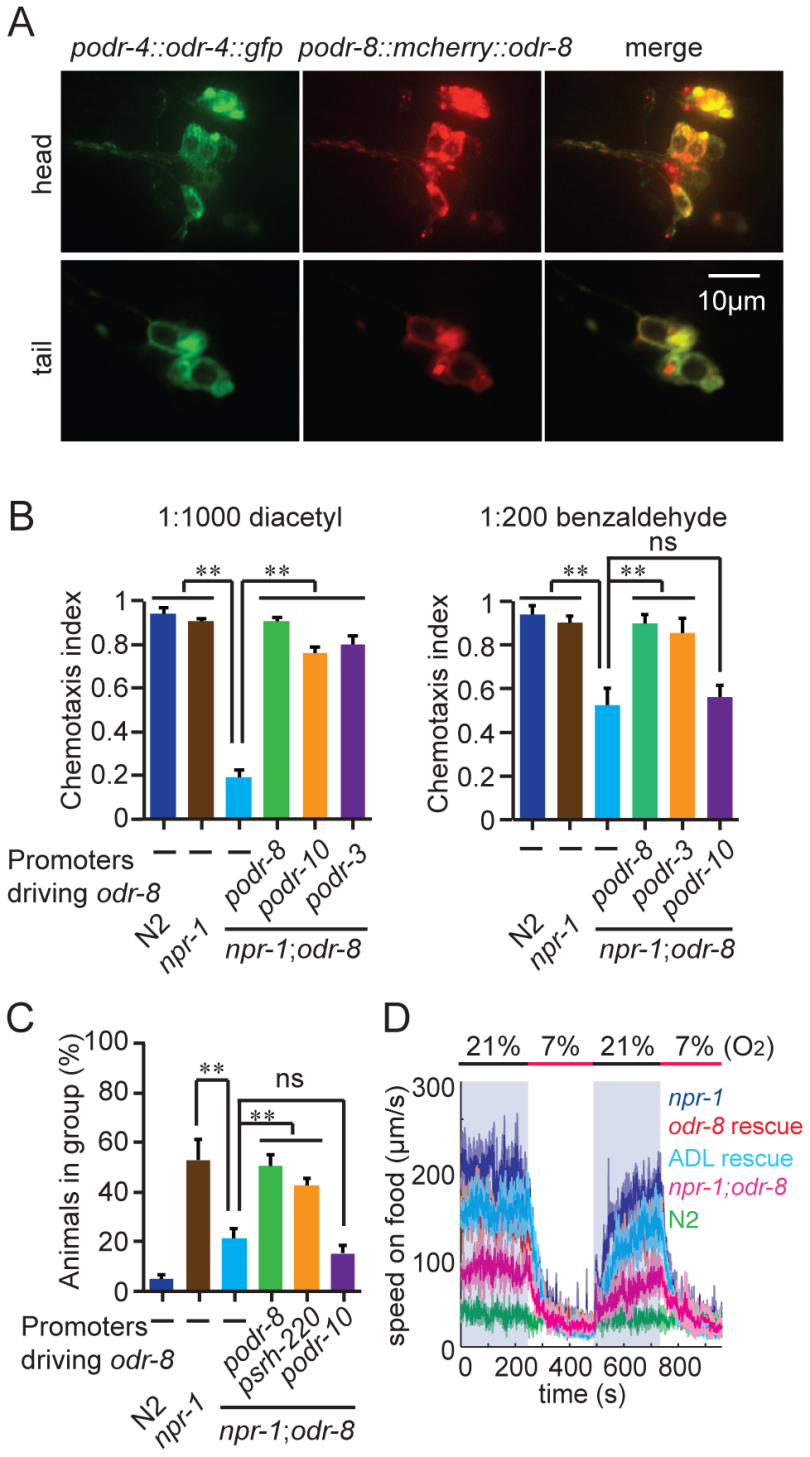
ODR-8 is co-expressed with ODR-4 in chemosensory neurons. (A) ODR-4 and ODR-8 are co-expressed in the same head and tail neurons. (B–D) Cell-specific rescue of *odr-8(ky31)* phenotypes. Like ODR-4, ODR-8 is required in AWA neurons for odortaxis to diacetyl (B), and in ADL neurons to sustain aggregation behavior (C) and high locomotory activity in 21% oxygen (D). The *podr-10* promoter drives expression only in the AWA neurons; *psrh-220* drives expression in ADL neurons; *podr-3* drives expression in AWA, AWB, AWC, ASH and ADF. ** *p*<0.01 (ANOVA, Bonferroni's multiple comparisons test) compared to non-transgenic controls.

The phenotypes of *odr-8* mutants closely resemble those of *odr-4* defective animals [20]. ODR-4 is a conserved tail-anchored transmembrane protein (Figure S3). To examine if *odr-4* or *odr-8* were expressed in the same cells, we made animals that carried both *podr-4::odr-4::gfp* and *podr-8::mCherry::odr-8* transgenes. Green and red fluorescence in these animals overlapped, consistent with ODR-4 and ODR-8 functioning in the same neurons (Figure 2A). We explicitly identified *odr-8* expressing neurons using the stereotyped position of *C. elegans* neurons and DiO dye-filling and a *podr-3::gfp* transgene as fiduciary markers. We observed mcherry-ODR-8 expression in 10 head neurons, the amphid neurons ADL, ASI, ASH, ASJ, ASG, ADF, ASK, AWA, AWB, AWC, and in two tail neurons, the phasmid neurons PHA and PHB. These are all the cells previously reported to express *odr-4* [20]. We did not detect expression in any additional neurons.

ODR-4 functions in the AWA neurons to promote chemotaxis to the odor diacetyl [20], and in the ADL neurons to promote aggregation [21]. Expressing *odr-8* specifically in AWA neurons, using the *odr-10* promoter, rescued *odr-8* odortaxis to diacetyl but not to benzaldehyde, which is sensed by the AWC neurons (Figure 2B). Similarly, expressing *odr-8* in ADL, using the *srh-220* promoter, restored aggregation behavior (Figure 2C) and oxygen responses (Figure 2D). Expressing *odr-8* from the *odr-3* promoter, which drives expression in the AWC olfactory neuron and a small number of other neurons including AWA, rescued *odr-8* chemotaxis to both benzaldehyde and diacetyl (Figure 2B). These data indicate that ODR-8 and ODR-4 function in the same neurons to promote specific behavioral responses.

### Olfactory receptors are retained in the ER in *odr-4* and *odr-8*

**Figure 3:**
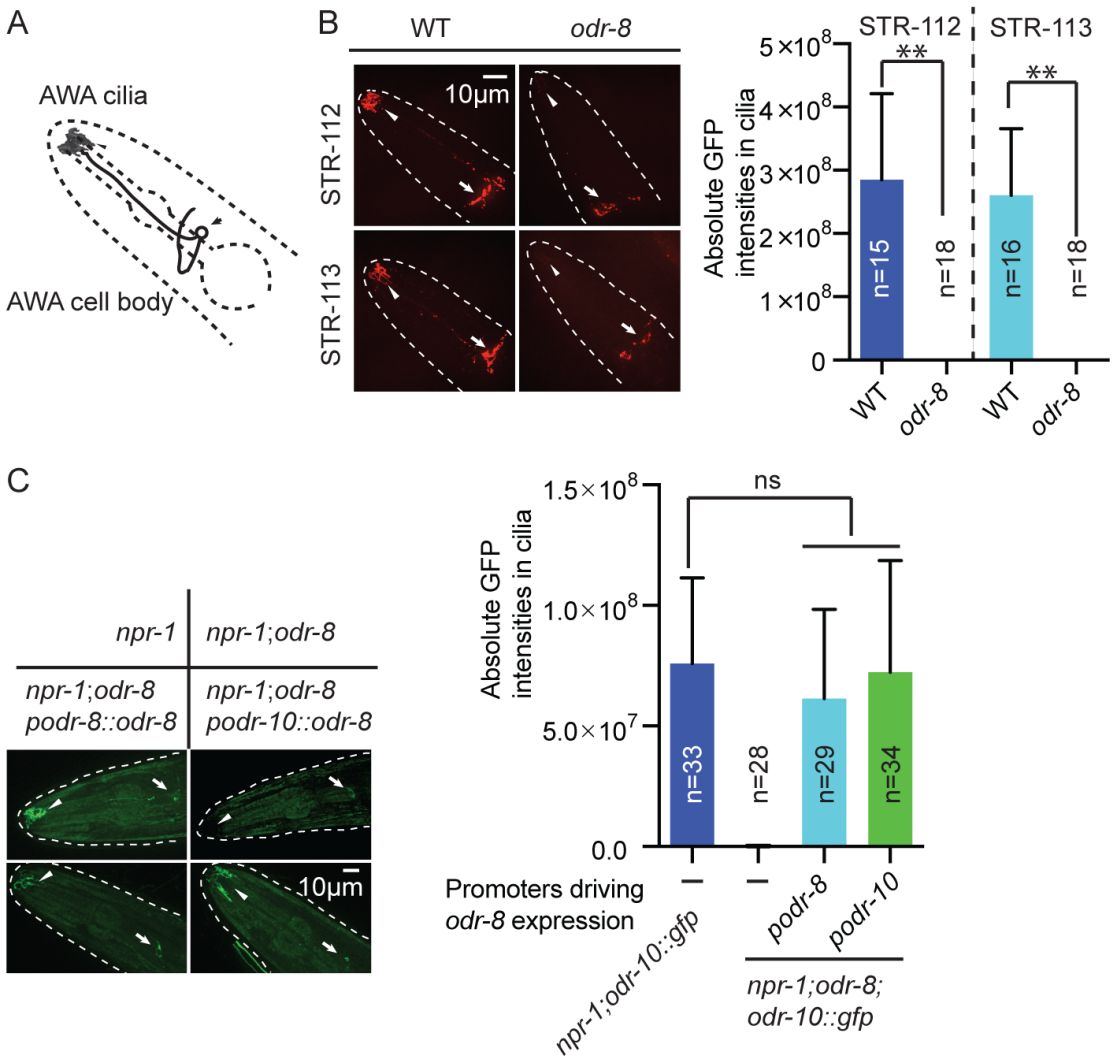
ODR-8 is required for the GPCR trafficking to the AWA cilia. (A) Schematic drawing of the *C. elegans* head with the arrow indicating the AWA cell body and the arrowhead pointing to the AWA cilia. (B) ODR-8 is required to localize GFP-tagged STR-112 and STR-113 odorant receptors to AWA cilia. Cilia localization in wild type and *odr-8* mutants is quantified to the right of the panel. (C) ODR-8 acts cell-autonomously to permit localization of ODR-10-GFP to the cilia of the AWA neurons. **, *p*<0.01 (ANOVA, Bonferroni's multiple comparisons test).

The behavioral defects of *odr-8* and *odr-4* mutants appear to reflect failure in the localization of a subset of G-protein coupled receptors (GPCR) to sensory cilia [20]. ODR-10 is an olfactory GPCR that mediates attraction to diacetyl [19]. In wild type animals ODR-10-GFP is localized to the sensory cilia of AWA olfactory neurons. In *odr-4* or *odr-8* mutants, ODR-10-GFP is predominantly found in unidentified endomembrane compartments [20]. We observed similar defects when we compared localization of two other GPCRs of the STR olfactory receptor family, STR-112 and STR-113, in the AWA neurons of wild type and *odr-8* mutants (Figure 3A–B and Figure S4).

To confirm that defects in *C. elegans* UfSP2 caused the ODR-10-GFP trafficking phenotype, we performed transgenic rescue experiments. ODR-10-GFP was correctly localized to AWA cilia in *odr-8* mutants bearing the F38A5.1 transgene, whereas in non-transgenic siblings ODR-10-GFP was localized to the cell body (Figure 3C). Expressing the F38A5.1a open reading frame specifically in the AWA neurons restored cilia localization of ODR-10-GFP, whereas expression in the ADL neurons did not, suggesting that ODR-8 functions cell-autonomously (Figure 3C and data not shown).

**Figure 4:**
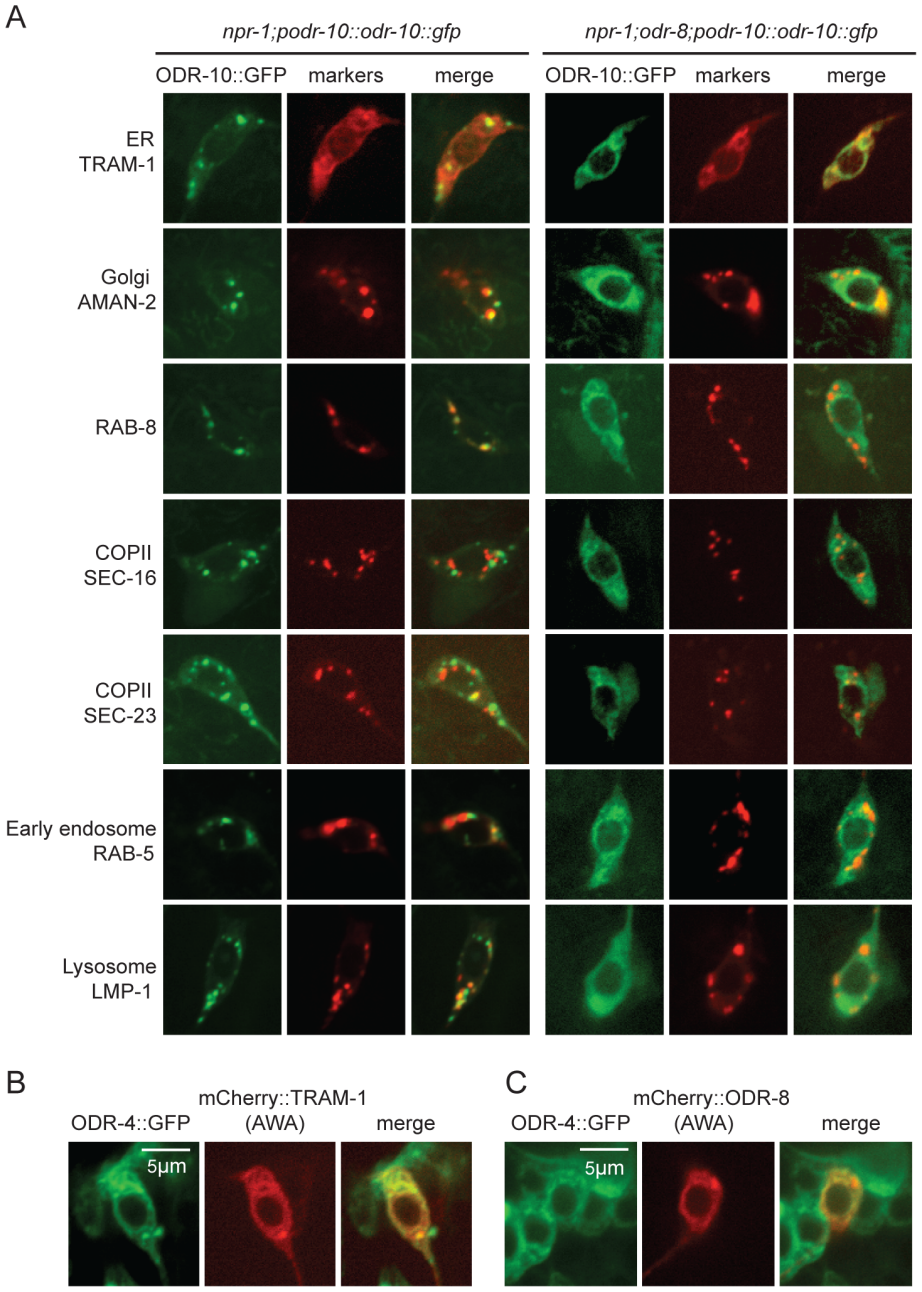
ODR-10-GFP is retained in the ER in *odr-8* mutants. (A) ODR-10-GFP co-localizes predominantly with RAB-8 and LMP-1 markers in wild type animals, but with ER markers in *odr-8(ky31)* mutants. (B–C) ODR-4 co-localizes with the ER marker TRAM-1 (B), and ODR-4 and ODR-8 partially co-localize with each other (C).

The small size of *C. elegans* neurons makes sub-cellular localization of fluorescently-tagged proteins challenging. Nevertheless, to investigate the fate of ODR-10-GFP in *odr-4* and *odr-8* mutants, we co-localized it with mCherry-tagged markers for different membrane compartments that we expressed specifically in AWA (Figure 4A). We included in our studies the rough ER marker TRAM-1, the Golgi marker alpha-mannosidase II [28], the ER exit site markers SEC-16 and SEC-23 [29], the early endosome marker RAB-5 [30], the lysosomal marker LMP-1 [31] [32], and RAB-8 which in sensory neurons marks vesicles destined for traffic to the cilia [33]. As expected, in wild type animals, ODR-10-GFP was predominantly localized to cilia, with some fluorescence in the cell body (Figure 4A and Figure S5). The cell body fluorescence co-localized most extensively with mCherry-RAB-8, in foci that were closely apposed to Golgi (Figure 4A and Figure S5). We also observed some co-localization with the lysosomal marker LMP-1 (Figure 4A and Figure S5). Co-localization of ODR-10-GFP with mCherry-RAB-8 has been reported previously when the receptor was expressed heterologously in the AWB and phasmid neurons [33]. In *odr-8* mutants co-localization of ODR-10-GFP and mCherry-RAB-8 was lost; instead ODR-10-GFP co-localized most extensively with markers for the ER (Figure 4A and Figure S5). These data suggest that in *odr-8* mutants the ODR-10-GFP receptor either does not exit the ER, or undergoes retrograde traffic back to the ER, or is degraded following ER exit.

### ODR-4 and ODR-8 proteins localize to the ER

We sought to establish the sub-cellular compartments to which ODR-4 and ODR-8 proteins predominantly localized to in AWA neurons. We made transgenes expressing ODR-4-GFP or mCherry-ODR-8 in AWA, and crossed them into animals in which different compartments were highlighted in AWA neurons using mCherry-tagged markers. ODR-4-GFP co-localized with the ER marker TRAM-1 (Figure 4B), suggesting ODR-4 functions in this compartment. mCherry-ODR-8 showed some co-localization with ODR-4, although we also observed diffuse staining consistent with cytoplasmic localization (Figure 4C).

### Disrupting AP-1 does not alter ER retention of ODR-10-GFP in *odr-8* mutants

**Figure 5:**
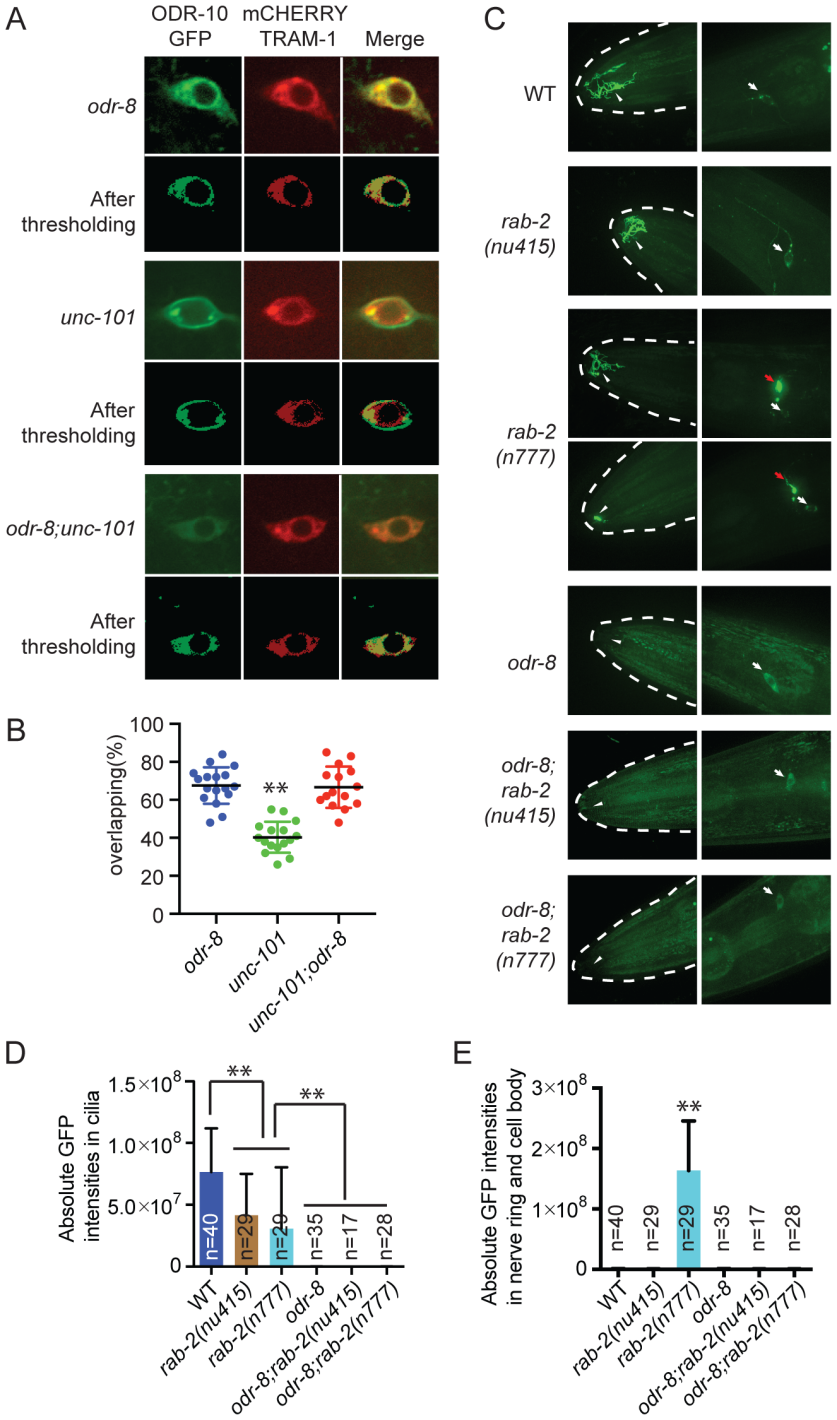
ODR-8 acts in an early step in ODR-10 trafficking to the AWA cilia. (A) Localization of ODR-10-GFP in AWA neurons of *odr-8(ky31)*, *unc-101(m1)* and *odr-8(ky31); unc-101(m1)* mutants. Thresholded images were obtained as described in the methods. (B) Quantitative analysis of ODR-10 and TRAM-1 co-localization in AWA neurons of *odr-8(ky31)*, *unc-101(m1)* and *odr-8(ky31); unc-101(m1)* mutants. (C). Localization of ODR-10-GFP in AWA neurons in *rab-2* mutants. Arrowheads indicate the AWA cilia, white arrows point to the AWA cell body and red arrows show the AWA processes in the nerve ring. In *rab-2(n777)* mutants, 23 out of 29 animals displayed normal ODR-10-GFP localization in AWA cilia, and 6 out of 29 show the ODR-10-GFP localization defects in AWA cilia. (D–E). Quantification of ODR-10-GFP signals in the cilia (D) and axons (including cell body) (E) of AWA neurons. **, *p*<0.01 (ANOVA, Bonferroni's multiple comparisons test); for E, *rab-2(n777)* shows *p*<0.01 compared to all other genotypes plotted.

We sought to design genetic experiments that shed light on where ODR-4 and ODR-8 function in the secretory pathway. Previous work indicates that post-Golgi sorting of ODR-10-GFP involves formation of clathrin-coated vesicles via adapter complex 1 (AP-1) [33]–[34]. Mutants lacking *unc-101*, which encodes the mu1 subunit of AP1, fail to traffic ODR-10-GFP to sensory cilia and instead traffic the receptor to the cell membrane. We reasoned that if *odr-8* mutations disrupt ODR-10-GFP receptor maturation prior to its reaching the Golgi, then disrupting *unc-101* should not alter ODR-10-GFP localization in *odr-8* mutants. Consistent with this, we observed no difference in ODR-10-GFP localization between *odr-8* and *odr-8; unc-101* animals (Figure 5A–B).

To extend our studies we imaged ODR-10-GFP localization in animals defective in or expressing dominant negative or dominant active version of the RAB GTPases RAB-1, RAB-2, RAB-6.1 and RAB-6.2. These RABs play roles at different steps in the secretory pathway [35], and some have previously been implicated in GPCR traffic [36]. In yeast, Rab1 is thought to be important for ER exit and traffic to the Golgi, Rab2 is implicated in retrograde traffic to the ER from the Golgi, and Rab6 plays a role in intra-Golgi traffic. For *rab-1*, *rab-6.1 and rab-6.2* we expressed dominant negative and dominant active transgenes in AWA using the *odr-10* promoter (see methods). For *rab-2* we used the deletion allele *nu415*, which is predicted to be null, and the *n777* allele, which is associated with a S149F mutation that constitutively activates RAB-2 [37]. None of the perturbations of RAB protein function completely blocked traffic of ODR-10-GFP to AWA cilia in wild type animals, although there were clear and significant reductions of ODR-10 cilia localization in all cases except when we expressed rab-6.1(Q70L) (Figure S6). In *rab-2(n777)* mutants, ODR-10 was not correctly localized to AWA cilia in 6 out of 29 animals (Figure 5C–E), and more strikingly, a significant amount of ODR-10 was mis-sorted to axons (Figure 5C–E). Previous work in *C. elegans* has implicated RAB-2 in biogenesis of dense core vesicles [37], [38] and in post-endocytic trafficking [39], suggesting a more complex role for this RAB protein. Axonal mislocalization of ODR-10 was not observed in *rab-2(n777); odr-8* double mutants, consistent with *odr-8* being required early in the secretory pathway (Figure 5C–E). For the remaining *rab* gene perturbations, *odr-8; rab* double mutants, and *odr-8* animals expressing mutant *rab* transgenes resulted in an ODR-10-GFP localization phenotype that was indistinguishable from that of *odr-8* mutants. Together, these observations and the *unc-101* data suggest that ODR-8 functions at an early step in GPCR trafficking.

### Disrupting *ufm-1* does not alter ODR-10-GFP localization in AWA neurons

**Figure 6:**
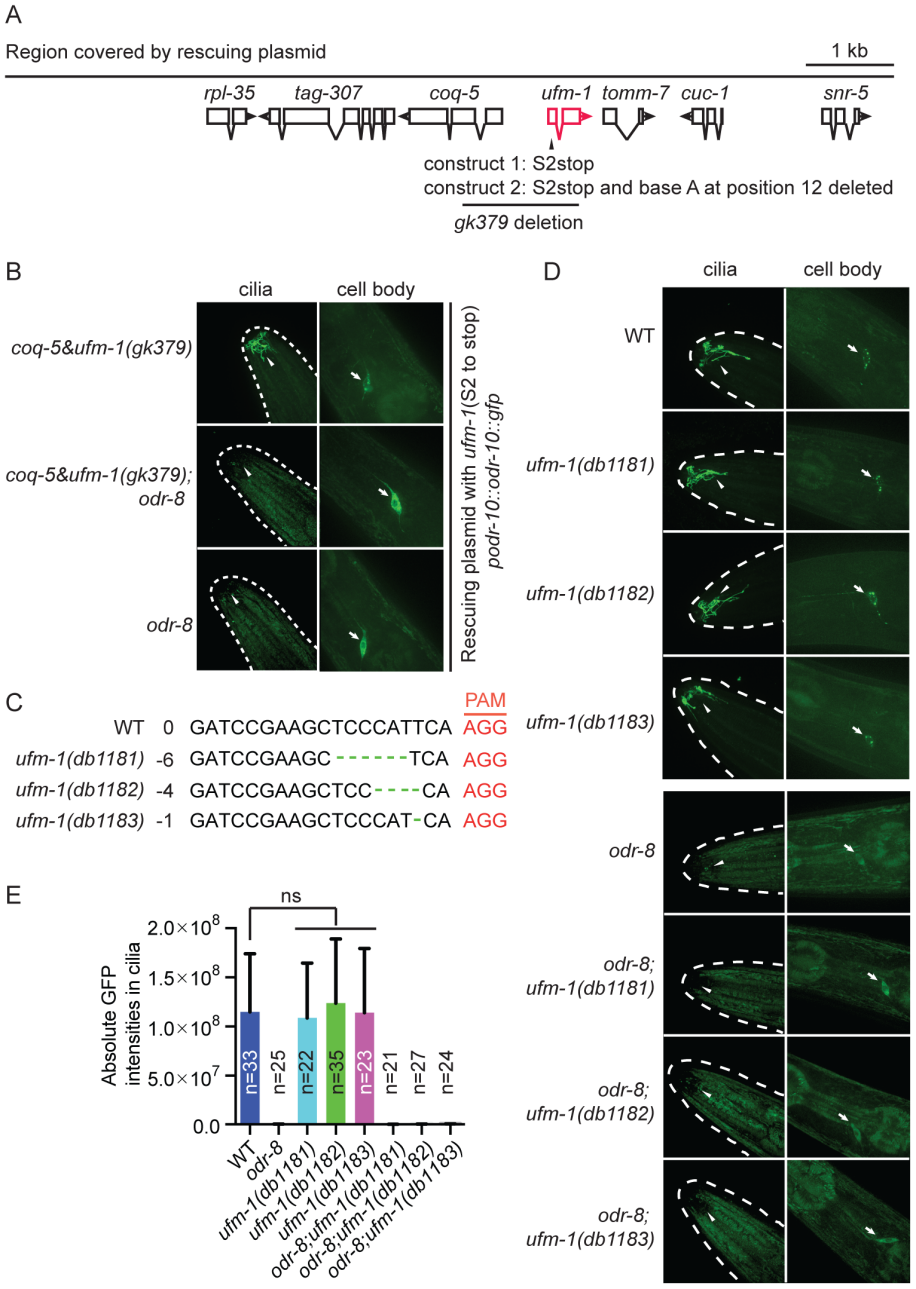
Deleting *ufm-1* does not alter ODR-10 localization. (A) Molecular nature of *ufm-1(gk379)* deletion allele. The *gk379* deletion disrupts two adjacent operons – one comprising *ufm-1* and *tomm-7* and one including *coq-5* and *tag-307*. *gk379* homozygous animals die as embryos or young larvae. To score the ODR-10-GFP localization in adult animals lacking *ufm-1* we rescued the *gk379* lethal phenotype using a 9 kb genomic fragment in which the *ufm-1* open reading frame is interrupted by a stop codon or by a combination of a stop codon and a frameshift mutation, as indicated. (B) Disrupting *ufm-1* does not change the ODR-10-GFP phenotype either in wild type animals or *odr-8(ky31)* mutants. (C) Mutations generated by CRISPR-Cas in the *ufm-1* gene. PAM refers to the protospacer adjacent motif. (D–E) Mutations in *ufm-1* do not block ODR-10 trafficking to AWA cilia in wild type animals, or rescue the ODR-10 localization defect in an *odr-8* mutant background. The ODR-10 cilia localization in (D) was quantified in (E).

Biochemical studies indicate that mouse UfSP2 de-ufmylase can activate pro-Ufm1 and cleave it from native protein conjugates [26] [25]. We therefore examined whether disrupting the *C. elegans* orthologs of Uba5 or Ufc1, the only known E1 and E2 ligases implicated in ufmylation [23] (Figure S1), or deleting *C. elegans ufm-1* itself, altered ODR-10 localization. *C. elegans* UBA-5 is encoded by T03F1.1 [40] whereas *C. elegans* UFC-1 is encoded by C40H1.6. UFM-1 is encoded by *tag-277*. Deletion mutations that are likely to be complete loss-of-function alleles are available for all three genes [40] (Figure S7A and Figure 6).

If the mutant phenotypes of *odr-8/ufsp2* reflected a failure to activate pro-*ufm1*, then disrupting ufmylation should recapitulate *odr-8* phenotypes. Conversely, if the primary defect of *odr-8/ufsp2* is failure to cleave UFM-1 from native protein conjugates, then disrupting *ufm-1* or inhibiting ufmylation might suppress *odr-8* phenotypes. Deleting *Ce-uba-5* or *Ce-ufc-1* did not affect viability, and had no visible effect on diacetyl chemotaxis or on ODR-10-GFP localization at AWA cilia in an otherwise wild type background (Figure S7B–C). No change in the phenotype of an *odr-8* mutant was observed upon further deletion of *Ce-uba-5* or *Ce-ufc-1* (Figure S7B–C). The only available allele that deletes *Ce-ufm-1, gk379*, also removes part of an operon that includes the ubiquinone biosynthetic enzyme *coq-5*, and results in embryonic lethality (Figure 6A). We suspected that lethality reflected disruption of the *coq-5* operon, rather than loss of *ufm-1*. Consistent with this, we could rescue the *gk379* embryonic lethality with a 9.1 kb genomic fragment of the region in which we truncated the *ufm-1* open reading frame by a premature stop codon or a frame shift mutation (Figure 6A). This allowed us to examine ODR-10-GFP localization at AWA cilia in adult animals lacking UFM-1. We did not observe any significant change in ODR-10-GFP localization, either in *odr-8(+)* or in *odr-8(−)* animals that also lacked *ufm-1* (Figure 6B). Furthermore, we could not detect UFM-1 expression in AWA neurons (Figure S7D–E).

To confirm our observations, we generated three independent *ufm-1* loss-of-function mutations using CRISPR-Cas gene-editing (Figure 6C) [41]. Two mutations caused frameshifts of the *ufm-1* open reading frame, and presumably are null alleles (Figure 6C). None of the mutations disrupted ODR-10-GFP cilia localization in *odr-8(+)* animals, or rescued the ODR-10-GFP cilia localization defect in an *odr-8* mutant background (Figure 6D–E). These data suggest that the primary role of ODR-8 UfSP2 in biogenesis of ODR-10-GFP is independent of ufmylation.

### ODR-8 UfSP2 functions through a non-catalytic mechanism

**Figure 7:**
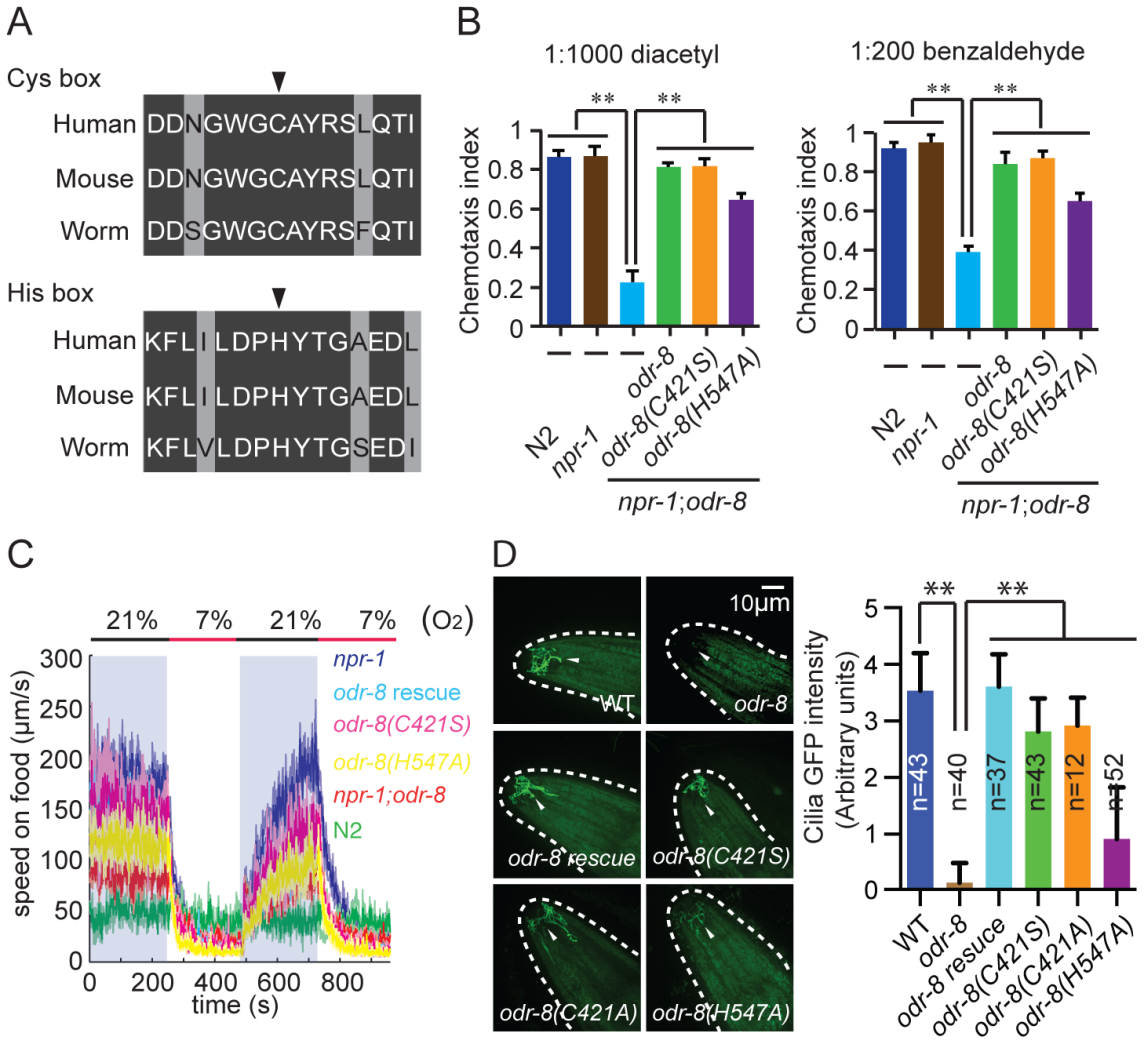
Non-catalytic action of ODR-8 Ufm1-specific protease 2. (A) Conservation of Cysteine and Histidine boxes in UfSP2 from worm, mouse and man. The catalytic cysteine and histidine residues are marked by an arrowhead. (B–D) *odr-8* transgenes in which the catalytic cysteine or histidine residues are mutated retain the ability to rescue *odr-8(ky31)* mutant phenotypes for odorant responses to diacetyl and benzaldehyde (B), oxygen responses (C) and biogenesis and localization of ODR-10 GPCR in AWA neurons (D). In D, ODR-10 localization in AWA cilia was scored using a scale from 0 to 4, where 0 reflects no observable localization and 4 reflects strong ODR-10 accumulation. Representative images for each genotype are shown. The *odr-8* allele rescued in these assays was *odr-8(ky31)*. ** indicates *p*<0.01 (ANOVA, Bonferroni's multiple comparisons test).

Biochemistry and crystallography have highlighted the active sites of the UfSP1 and UfSP2 proteases [25]–[27]. Like in several de-ubiquitinases, the active sites of these enzymes include highly conserved cysteine and histidine boxes (Figure 7A). The cysteine box contains the catalytic cysteine residue that is thought to undergo de-protonation, enabling nucleophilic attack of the carbonyl carbon of incoming substrates. The histidine in the histidine box assists this de-protonation. *In vitro* biochemical studies show that mutating the active site cysteine residue to a serine, or the histidine to an alanine, abolishes UfSP1 and UfSP2 proteolytic activity [25], [27].

To examine if an intact active site was required for ODR-8/UfSP2 *in vivo* functions, we made a transgene encoding an ODR-8 in which the active site cysteine was mutated to a serine. Unexpectedly, this transgene rescued all the behavioral defects of *odr-8* mutants we tested, and restored ODR-10-GFP localization to AWA cilia (Figure 7B–D). Since serine residues can engage in nucleophilic attack, we also created a transgene encoding an ODR-8 in which the active site cysteine was mutated to an alanine. This Cys-to-Ala active site mutant also rescued the behavioral and cell biological defects of *odr-8* mutants (Figure 7D and data not shown). We next mutated the active site histidine residue to an alanine, and tested the biological function of the protein in transgenic animals. The His-to-Ala *odr-8* transgene also substantially although not completely rescued the behavioral and cell biological phenotypes of *odr-8* mutants. Incomplete rescue could be due to effects of this mutation on protein structure (Figure 7B–D). Together, these data suggest that the protease activity of ODR-8 UfSP2 is not essential for its *in vivo* function in GPCR maturation.

### ODR-4 and ODR-8 form an ER complex that promotes GPCR trafficking

**Figure 8:**
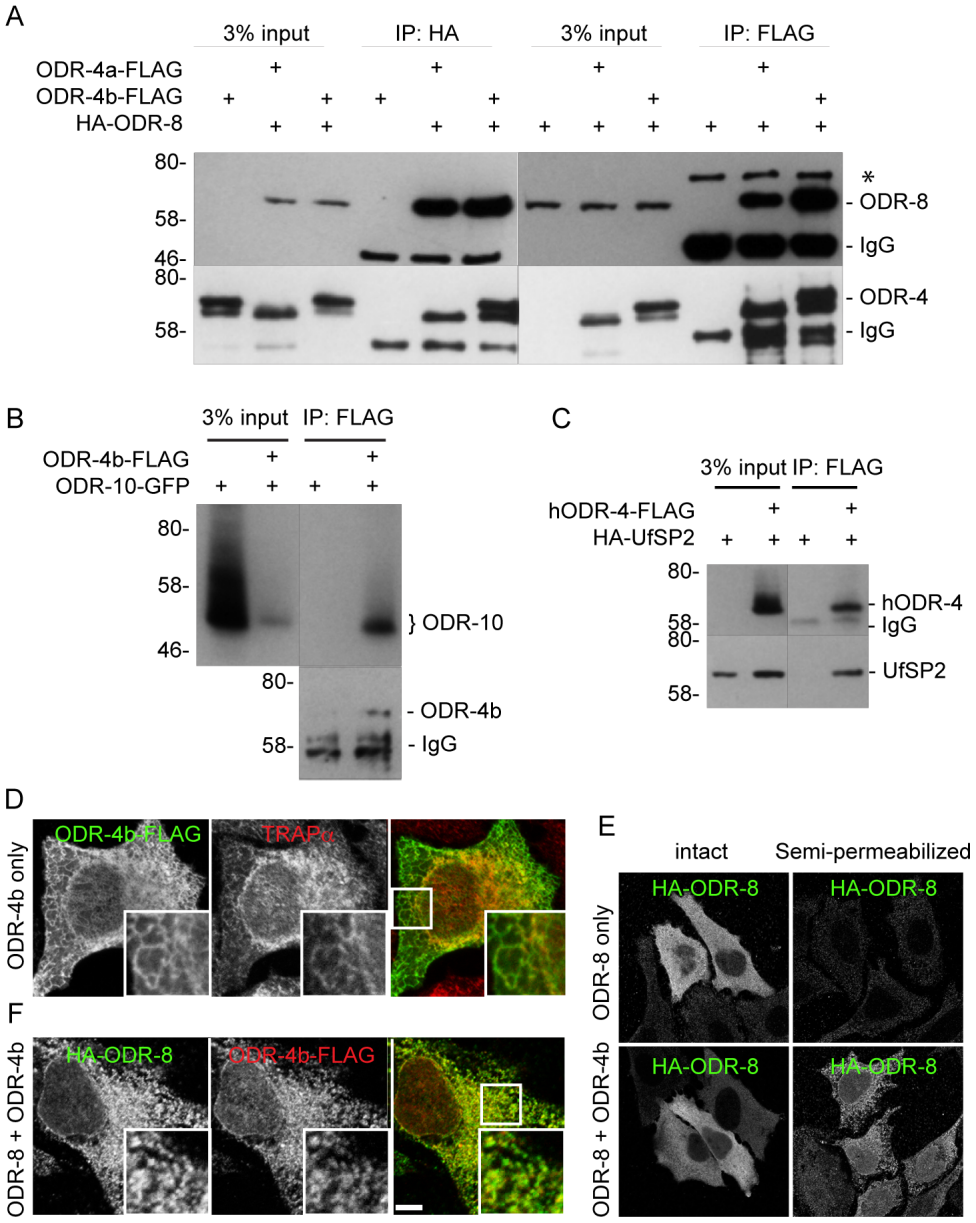
ODR-4, ODR-8 and ODR-10 form a complex at the ER. (A) HEK293 cells were co-transfected with ODR-4a-Flag, ODR-4b-FLAG and HA-ODR-8, and cell lysates were subjected to immunoprecipitation using indicated antibodies. The resulting precipitates were examined by immunoblot analysis with mouse anti-FLAG and mouse anti-HA antibodies. The asterisk indicates a non-specific band. (B) HEK293 cells were co-transfected with ODR-10-GFP with or without ODR-4b-FLAG, and cells lysates were subjected to immunoprecipitation using mouse anti-HA and rabbit anti-GFP antibodies. (C) HEK293 cells were co-transfected with human ODR4-FLAG and human HA-UfSP2, and cell lysates were subjected to immunoprecipitation using a FLAG antibody. The resulting precipitates were examined by immunoblot analysis with mouse anti-FLAG and mouse anti-HA antibodies. (D) HeLa cells were transiently transfected with ODR-4b-FLAG for 3 days. After fixation, the cells were subjected to immunocytochemistry using mouse anti-Flag and rabbit anti-TRAPα antibodies. (E) HeLa cells co-transfected with HA-ODR-8 and ODR-4b-FLAG as in panel D. fixed, and stained using rabbit anti-Flag and mouse anti-HA antibodies. Bar, 5 µm. (F) HeLa cells were transiently transfected with HA-ODR-8 alone or with HA-ODR-8 and ODR-4b-FLAG and cultivated for 3 days. In the panel marked ‘semi-permeabilized’ cells were treated with digitonin for 5 min prior to fixation, whereas in the panel labeled ‘intact’ the digitonin in step was omitted. Immunocytochemistry was performed using rabbit anti-Flag and mouse anti-HA antibodies.

The expression patterns of ODR-8 and ODR-4, the subcellular localizations of the two proteins, and the phenotypes displayed by *odr-8* and *odr-4* mutants all suggested that these two proteins function together to promote ODR-10 trafficking to the cilia. However, the small size of AWA neurons and the limits of light microscopy prevented high-resolution co-localization of ODR-4 and ODR-8. In addition, biochemical analyses of ODR-4/ODR-8/ODR-10 protein interactions in worm extracts were precluded by the restricted expression patterns of these proteins *in vivo*. To circumvent these obstacles, we heterologously expressed ODR-4, ODR-8, and ODR-10 in cultured mammalian cells and analyzed their biochemical interactions and sub-cellular localization. Epitope-tagged ODR-8 and ODR-4 (two isoforms, ODR-4a and ODR-4b) were expressed either individually or in combination in HEK293 cells and analyzed by immunoprecipitation (IP). HA-ODR-8 co-precipitated with either ODR-4a-FLAG or ODR-4b-FLAG, regardless of which component was subjected to IP (Figure 8A). Immunoprecipitation of ODR-4 could also co-precipitate ODR-10-GFP (Figure 8B), while ODR-8 did not seem to directly interact with ODR-10 (Data not shown). Since ODR-4 and ODR-8 are conserved, we asked if their human orthologs also interacted biochemically. Immunoprecipitation of hODR4-FLAG brought with it HA-UfSP2 (Figure 8C), suggesting that like the *C. elegans* proteins, the human orthologs can form a complex in human cells.

Immunofluorescence studies localized the site of these interactions to the ER. Here, we found that ODR-4 co-localized with the ER resident protein TRAPα (Figure 8D). By contrast, ODR-8 was diffusely cytosolic when expressed alone, but somewhat reticular when co-expressed with ODR-4 (Figure 8E). This suggested that ODR-8 was recruited to the ER via its physical interaction with ODR-4. This was confirmed by demonstrating that ODR-8 was efficiently released from cells by permeabilization of the plasma membrane, but partially retained when co-expressed with ODR-4 (Figure 8F). The retained ODR-4 and ODR-8 were co-localized, consistent with their physical interaction (Figure 8E). Thus, ODR-4 and ODR-8 form a physical complex at the ER membrane.

ODR-10-GFP was also ER-localized in HeLa cells (Figure S8A). This localization remained qualitatively similar in cells co-expressing ODR-4 and/or ODR-8 (Figure S8B). This suggests that while ODR-4 and ODR-8 are clearly necessary for proper ODR-10 surface trafficking in AWA neurons, they may not be sufficient in the heterologous HeLa cell system. This remains to be investigated further. Nevertheless, the analysis in cells, together with the genetic and (lower resolution) localization data in AWA neurons, indicates that ODR-4 and ODR-8 form an ER-localized complex that facilitates ODR-10 surface expression.

## Discussion

ODR-8, the *C. elegans* ortholog of Ufm1 specific protease 2, is involved in the biogenesis of GPCRs via a non-catalytic mechanism that does not require Ufm1. *odr-8*/UfSP2 mutants, like *odr-4* mutants, fail to localize a subset of olfactory receptors to sensory cilia. This defect occurs at an early step in secretory pathway, most likely prior to exit from the endoplasmic reticulum. Consistent with this, ODR-4, ODR-8 and ODR-10 form a complex in the ER of HEK cells.

UfSP2 and its relative UfSP1 were first identified biochemically in mouse by virtue of their ability to process the ubiquitin-like protein Ufm1 [26]. UfSP2 is found throughout eukaryotes, from plants to man. UfSP1 is found in mouse, man, and flies but not *C. elegans*. Crystallography shows that UfSP1 and UfSP2 have a papain-like domain with a Cysteine-Aspartate-Histidine catalytic triad [25], [27]. The fold structure is most similar to that of the autophagy gene Atg4, which de-conjugates Atg8 ubiquitin-like proteins from phosphatidylethanolamine and promotes autophagosome formation. Mutagenesis of active site residues confirms the importance of these residues for *in vitro* UfSP protease activity: UfSP1 and UfSP2 are catalytically dead if the catalytic cysteine is mutated to a serine. The residues comprising the papain fold, including the catalytic residues, are highly conserved across UfSP orthologs, with 40% identity from grape vine to man.

Unexpectedly, we find that active site residues of ODR-8 UfSP2 are not required for any of its known *in vivo* functions. Mutating the catalytic cysteine or histidine residues of *C. elegans* UfSP2 does not abolish these functions. Thus, at least in *C. elegans*, and perhaps more generally, the major functions of UfSP2 do not require proteolytic activity. In addition, deleting *Ce-ufm1* does not perturb localization of the ODR-10-GFP GPCR in AWA neurons, either in wild-type or in *odr-8* mutants. This suggests that ODR-8 UfSP2 and UFM-1 do not necessarily have to function together *in vivo*.

ODR-4 localizes predominantly to the ER *in vivo*, and the soluble ODR-8 UfSP2 protein also shows some ER localization. Previous work showed that mouse UfSP2 is recruited to the ER when expressed with its client protein C20orf116 [25]. We show that ODR-4 can also recruit ODR-8 UfSP2 to the ER in HeLa cells. Disrupting either ODR-4 or ODR-8 results in some olfactory GPCRs, including ODR-10, being retained in the ER rather than trafficking in RAB-8-containing vesicles to sensory cilia. Neither ODR-4 nor ODR-8 accumulates preferentially at ER exit sites, and ODR-10-GFP does not appear to accumulate at ER exit sites in *odr-4* or *odr-8* mutants, suggesting these proteins are not involved in ER exit *per se*. ODR-4 and ODR-8, and ODR-4 and ODR-10 interact biochemically in HEK cells, A simple interpretation of all our data is that ODR-4 and ODR-8/UfSP2 form a complex in the ER with some olfactory receptors, including ODR-10, that allows these GPCRs to mature and become competent to exit the ER. In the absence of maturation, these GPCRs are retained in the ER. Quality control of folding in the ER is thought to involve cycles of ubiquitination and de-ubiquitination [42]–[44]. One highly speculative model is ODR-8/UfSP2 binds to ubiquitin or a ubiquitin-like molecule to regulate maturation of GPCRs.

In *C. elegans* ODR-4 and ODR-8/UfSP2 are expressed in the same small set of chemosensory neurons, and their function appears limited to maturation of a subset of GPCR chemoreceptors. Orthologues of ODR-8/UfSP2 and ODR-4 are found in organisms that do not have a nervous system, such as plants. Moreover, the mouse orthologs of ODR-4 and ODR-8 are expressed in many non-neuronal tissues. A simple explanation is that outside *C. elegans* these proteins participate in maturation of non-olfactory GPCRs. Interestingly, human ODR-4 and human UfSP2 form a complex in HEK cells, suggesting they also function together in humans.

## Methods

### Strains

Animals were grown under standard conditions [45]. Strains used are listed in Table S1.

### Behavioral assays

Aggregation behavior, chemotaxis, and responses to changes in oxygen levels were measured as described previously [46]–[48].

### Molecular biology

Whole genome sequencing was carried out on the Illumina HiSeq 2000 platform.

Plasmid construction: Plasmids were constructed using the multi-site Gateway system (Invitrogen). Promoters used include *podr-10* (1.2 kb), *podr-8* (3.1 kb), *psrh-220* (2.1 kb), *pstr-1*(4 kb), *podr-3* (2.7 kb), and *pufm-1*(4.1 kb); numbers refer to DNA upstream of the initiation codon. Promoters were cloned into the first position of the Gateway system, genes of interest were cloned at the second position, and the *unc-54* 3′UTR or the SL2::mCherry sequence at the third position. To rescue the *odr-8* mutant phenotypes, we used a genomic fragment encoding F38A5.1a (Figure S9). To generate in frame C-terminal GFP or mCherry fusions, genes of interest were cloned in the second Gateway position without a stop codon, and the third position was occupied by GFP or mCherry DNA. For constructs encoding N-terminus GFP or mCherry fusion proteins, GFP or mCherry sequences without stop codons were placed at the second position and genes of interest at the third position. When the third position was occupied by sequences other than *unc-54* 3′UTR, we used a destination vector that placed *unc-54* 3′UTR sequences downstream of the 3^rd^ Gateway insert. We rescued the lethality associated with the *gk379* deletion using a ∼9.1 kb genomic fragment centered on the *ufm-1* gene. This fragment was PCR amplified using primers flanked with attB1 and attB2 sites and cloned into pDonr221 using the BP reaction. To target the *ufm-1* gene by CRISPR, a *ufm-1* gene-specific sequence was cloned into the sgRNA vector bearing *rpr-1* promoter using the Gibson assembly kit, as described [41].

Site-specific mutagenesis: Mutagenesis was performed using the Quikchange II XL kit from Agilent. Changes made include: *odr-8(C421S: TGT to TCT)*; *odr-8(C421A: TGT to GCT)*; *odr-8(H547A: CAT to GCT)*; *ufm-1(S2stop: TCG to TAA)*; *ufm-1(S2stop:TCG to TAA; nucleotide A at position 12 deleted)*; *rab-1(GDP bound S25N: TCG to AAT)*; *rab-1(GTP bound Q70L: CAG to CTG)*; *rab-6.1(GDP bound T25N: ACT to AAT)*; *rab-6.2(GTP bound Q70L: CAG to CTG)*; *rab-6.2(GDP bound T24N: ACC to AAC)* and *rab-6.2(GTP bound Q69L: CAG to CTG)*.

Transgenic strains: the *ufm-1* rescue plasmid and fusion genes that express fluorescent markers of sub-cellular compartments were injected at 2–4 ng/ul together with 50 ng/ul of coelomocyte marker and 1 kb ladder. Other constructs were microinjected at 50 ng/ul.

### Microscopy

Animals were mounted on 2% agarose pads containing 50 mM sodium azide. Confocal images were taken on a Nikon Eclipse Ti inverted microscope coupled to the Andor Ixon EMCCD camera and spinning disk confocal unit. GFP signals were captured with a 100 ms exposure time at an EMCCD Gain of 300, except for the ODR-4-4 images, which were captured with 5 ms exposure times. The mCherry signals of subcellular markers were imaged with different exposure times, according to their signal intensities. The subcellular localization images were generated by averaging 32 images.

Co-localization of ODR-8 with various markers was quantified as described [39] with slight modifications. We used the co-localization analysis package in Huygens software (Figure S5). A region of interest was cropped around the AWA cell body. The pixel intensity representing the brightest 4% of pixels in each image was extracted for both green and red channels. The pixel intensity values corresponding to the cut-off of the brightest 4% of pixels were used to threshold each image. The thresholded images were used to calculate the percentage of co-localization between ODR-8 and different markers.

To quantify the amount of ODR-10::GFP in the cilia and cell body, z-stack images were taken on a spinning disk confocal microscope using a 100× lens and 100 ms exposure time. The 3-D images were reconstituted with the aid of IMARIS software (Figure S4). GFP pixel intensities brighter than 1200 were cropped by creating a surface with 0.25 µm details. The total pixel intensities inside the surface were calculated.

### CRISPR-Cas9 induced mutations

Frameshift mutations in *ufm-1* were obtained using the CRISPR-Cas9 system, as described [41]. Briefly, Cas9 was expressed from the *eft-3* promoter, while the sgRNA targeting the *ufm-1* gene was expressed from the *rpr-1* promoter. The Cas9 and sgRNA constructs were injected into the worm gonad with co-injection marker (cc::GFP). 38 ccGFP-positive F1 animals were singled, and genotyped after the eggs were laid.

### Immunofluorescence for mammalian cells

HeLa cells grown on coverslips were washed with PBS and fixed in 3.7% formaldehyde in PBS for 15 min. For semi-permeabilization, cells were treated with 50 µg/ml digitonin in KHM buffer (110 mM KAc, 20 mM HEPES (pH 7.4) and 2 mM MgCl_2_) for 5 min at 4°C before fixation. Fixed cells were permeabilized with 0.1% Triton ×100 in PBS for 5 min, blocked with 10% fetal bovine serum in PBS for 30 min, and incubated with primary antibodies for 1 h. After washing, cells were incubated with AlexaFluor 488-conjugated goat anti-mouse IgG and/or AlexaFluor 564-conjugated goat anti-rabbit IgG secondary antibodies (Invitrogen) for 60 min. Plasma membrane was stained using 0.01% PKH26, according to the manufacturer's instructions (Sigma). Images were acquired on a confocal laser microscope (LSM 780, Zeiss) using a 63× oil-immersion objective lens with a numerical aperture (NA) of 1.42.

### Immunoprecipitation and immunoblotting

Cell lysates were prepared in a lysis buffer (50 mM Tris-HCl (pH 7.5), 150 mM NaCl, 1% Triton X-100, 1 mM phenylmethanesulfonyl fluoride (PMSF) and protease inhibitor cocktail (complete EDTA-free protease inhibitor, Roche)). The lysates were clarified by centrifugation at 15,000 rpm and subjected to immunoprecipitation using anti-Flag M2 affinity gel and anti-HA agarose (Sigma). Precipitated immunocomplexes were washed five times in a washing buffer (50 mM Tris-HCl (pH 7.5), 150 mM NaCl, 1 mM EDTA, and 1% Triton X-100) and boiled in sample buffer. Samples were subsequently separated by SDS-PAGE and transferred to Nitrocellulose membranes (Biorad). Immunoblot analysis was performed with anti-GFP, anti-Flag M2 antibody and anti-HA (clone 16B12), and visualized with Super Signal West Pico Chemiluminescent substrate (Pierce).

## Supporting Information

Figure S1 The Ufm1 conjugation pathway. (A) The Ufm1 conjugation pathway highlighted to date by biochemistry [49]. Pro-Ufm1 is activated by UfSP1 or UfSP2 cysteine proteases. Mature Ufm1 is activated by the Uba5 E1 like enzyme, and then transferred to the E2 like enzyme Ufc1. The E3 like enzyme Ufl1 conjugates Ufm1 onto target substrate. Ufmylated substrates can be de-ufmylated by UfSP1 or UfSP2. (B) Alignment of Ufm1 sequences from different species, indicating where we generated frame shift mutations using CRISPR. Colors refer to amino acid types.
(TIF)

Figure S2 ODR-8 UfSP2 across phylogeny. (A) Alignment of UfSP2 from mouse, human, *C. elegans* and *A. thaliana*. Colors refer to amino acid types. (B) Phylogenetic tree of UFSP2/ODR-8 orthologues, showing that this protein is found in many eukaryotes, including sponges, plants and most metazoan phyla.
(TIF)

Figure S3 ODR-4 across phylogeny. (A) Alignment of ODR-4 from mouse, human, *C. elegans* and fish. Colors refer to amino acid types. (B) Phylogenetic tree of ODR-4 orthologues, showing that this protein, like UfSP2, is found in many eukaryotes.
(TIF)

Figure S4 Quantification of ODR-10-GFP localization in the cilia of AWA neurons. A region of interest (ROI) was cropped and thresholded with 1200 signal intensity. Pixels with intensities over 1200 were computed and summed.
(TIF)

Figure S5 Co-localization of ODR-10-GFP with various markers in AWA neurons. (A–D) Examples of co-localization analysis of ODR-10-GFP with different markers in wild type (A–B) and *odr-8* mutants (C–D). See Methods for details. (E–F). Quantification of ODR-10-GFP co-localization with different markers in wild type (E), and *odr-8* mutants (F). ** indicates *p*<0.01 by ANOVA, Bonferroni's multiple comparisons test.
(TIF)

Figure S6 ODR-10-GFP localization in mutants expressing defective *rab* proteins. (A) Localization of ODR-10-GFP in animals expressing defective *rab* proteins in AWA neurons of *npr-1* and *odr-8(ky31); npr-1* animals. For *rab-1*, *rab-6.1* and *rab-6.2*, the indicated dominant active and dominant negative versions of the RAB proteins were transgenically expressed in AWA from the *odr-10* promoter. For *rab-2*, we used the null allele *nu415* and the dominant active allele *n777* [37] [39]. (B) Quantitation of ODR-10-GFP accumulation in cilia in different *rab* backgrounds. **, *p*<0.01 (ANOVA, Bonferroni's multiple comparisons test).
(TIF)

Figure S7 Deleting *ufc-1* or *uba-5* does not alter ODR-10 function. (A) Molecular nature of mutations in *ufc-1* and *uba-5* deletion alleles. (B) *uba-5* and *ufc-1* deletion mutants show wild type chemotaxis to diacetyl. (C) Deleting *uba-5* does not alter the ODR-10-GFP phenotype in wild type or *odr-8* mutants. (D) ODR-10::GFP expression driven by the *ufm-1* promoter. (E) Expressing a *ufm-1::SL2::mCherry* operon from the *ufm-1* promoter does not highlight AWA neurons marked with ODR-10::GFP.
(TIF)

Figure S8 ODR-4 and ODR-8 are not sufficient to promote efficient ODR-10-GFP expression at the cell surface in HeLa cells. (A) HeLa cells were transiently transfected with ODR-10-GFP for 3 days. After fixation, the cells were subjected to immunocytochemistry by using rabbit anti- TRAPα antibodies, to highlight the ER. (B) HeLa cells were transiently transfected with indicated plasmids for 3 days. After fixation, plasma membrane was stained by PKH26. Bar, 5 µm.
(TIF)

Figure S9 Sequence of the pEXPRESSION vector used for *odr-8* mutant phenotype rescue. Green: *odr-8* promoter; yellow: *odr-8* genomic DNA; cyan: *odr-8* 3′UTR; grey: *unc-54* 3′UTR.
(PDF)

Table S1 *C. elegans* strains used in this study.
(DOCX)
