# Supplementary figures and images for: An ER Complex of ODR-4 and ODR-8/Ufm1 Specific Protease 2 Promotes GPCR Maturation by a Ufm1-Independent Mechanism
Source: PLoS Genet. 2014 Mar 6;10(3):e1004082. doi: 10.1371/journal.pgen.1004082 (PMC3945108; doi:10.1371/journal.pgen.1004082)

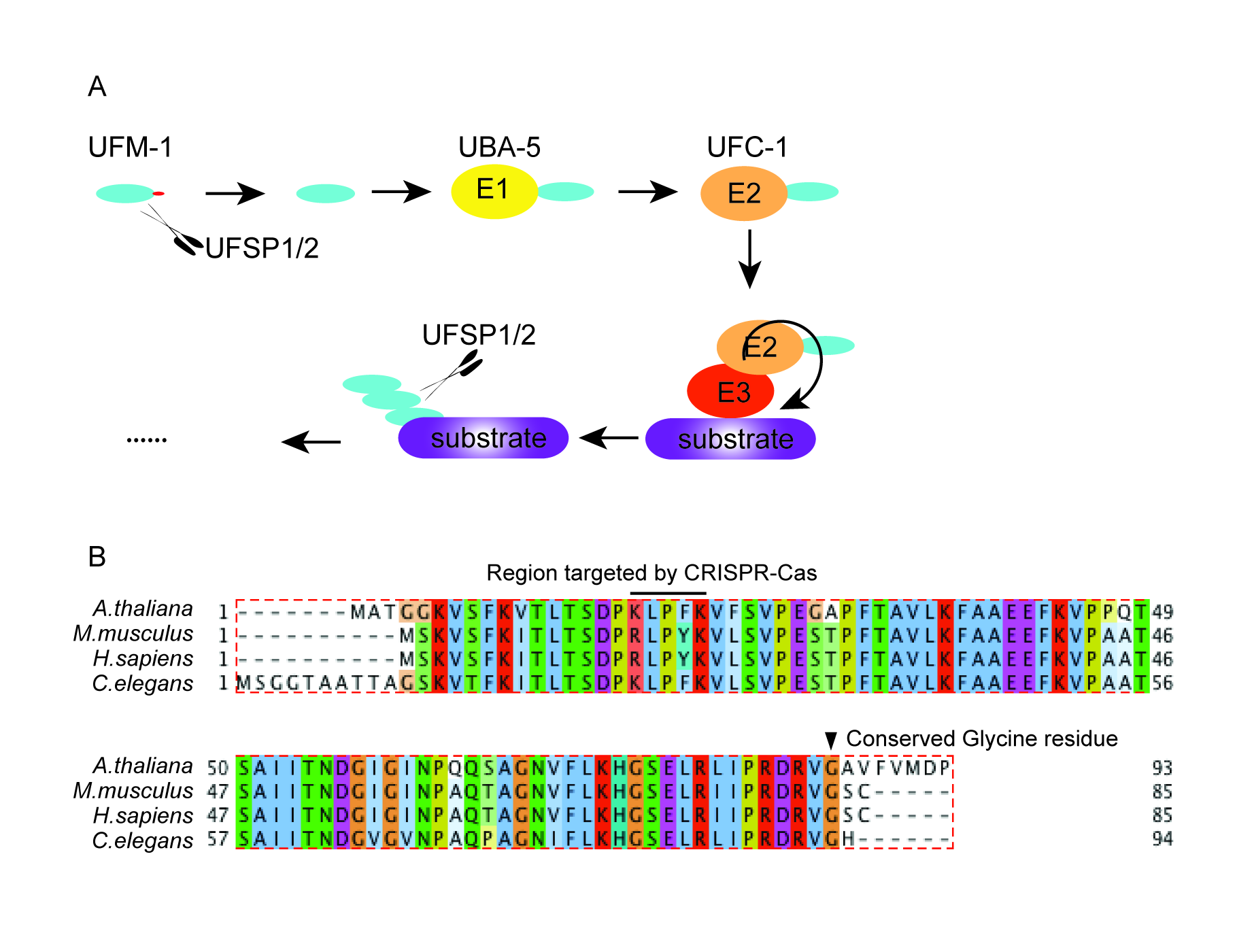

Supplement: Figure S1 — The Ufm1 conjugation pathway. (A) The Ufm1 conjugation pathway highlighted to date by biochemistry [49]. Pro-Ufm1 is activated by UfSP1 or UfSP2 cysteine proteases. Mature Ufm1 is activated by the Uba5 E1 like enzyme, and then transferred to the E2 like enzyme Ufc1. The E3 like enzyme Ufl1 conjugates Ufm1 onto target substrate. Ufmylated substrates can be de-ufmylated by UfSP1 or UfSP2. (B) Alignment of Ufm1 sequences from different species, indicating where we generated frame shift mutations using CRISPR. Colors refer to amino acid types. (TIF) [file pgen.1004082.s001.tif]

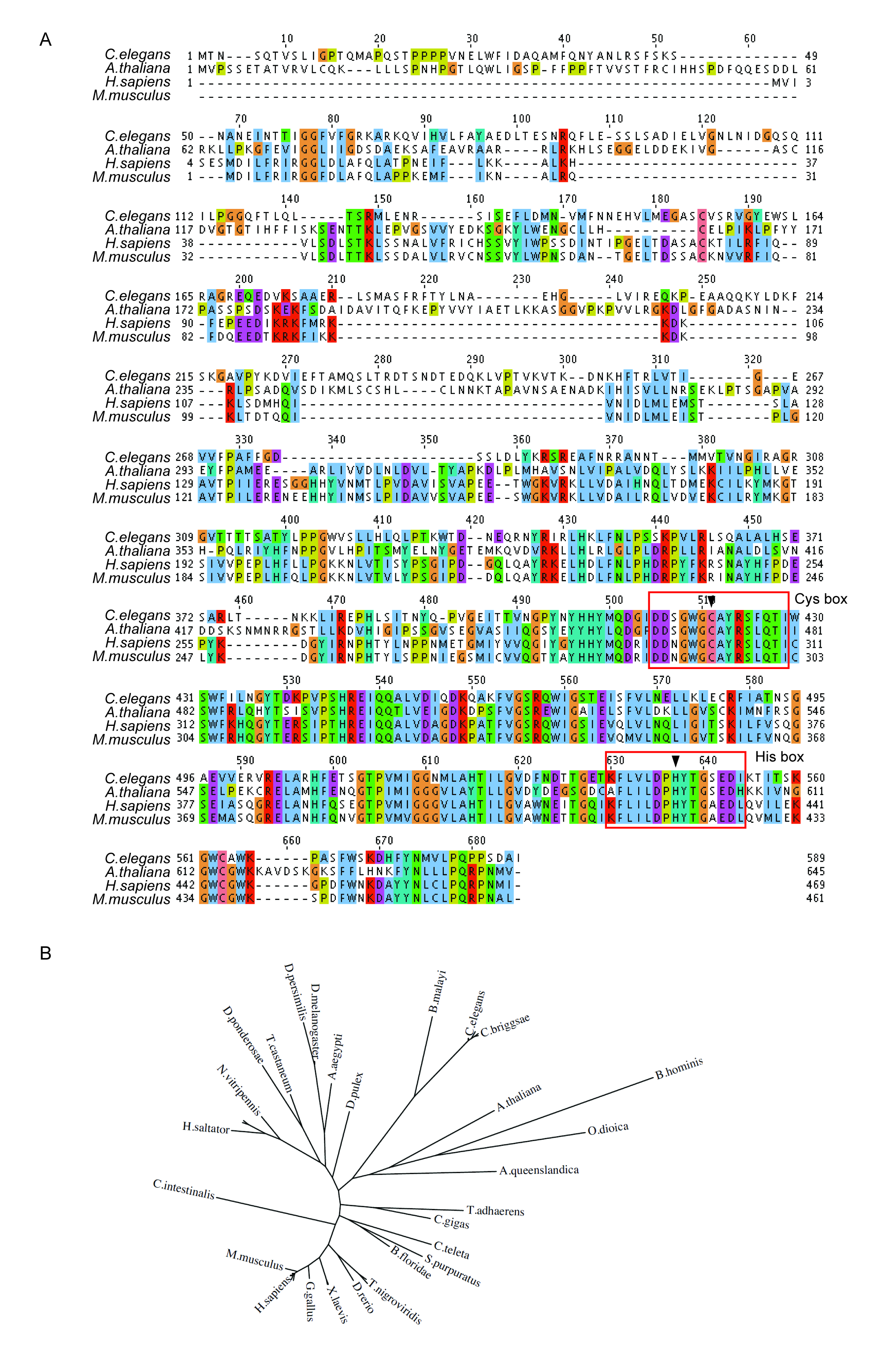

Supplement: Figure S2 — ODR-8 UfSP2 across phylogeny. (A) Alignment of UfSP2 from mouse, human, C. elegans and A. thaliana. Colors refer to amino acid types. (B) Phylogenetic tree of UFSP2/ODR-8 orthologues, showing that this protein is found in many eukaryotes, including sponges, plants and most metazoan phyla. (TIF) [file pgen.1004082.s002.tif]

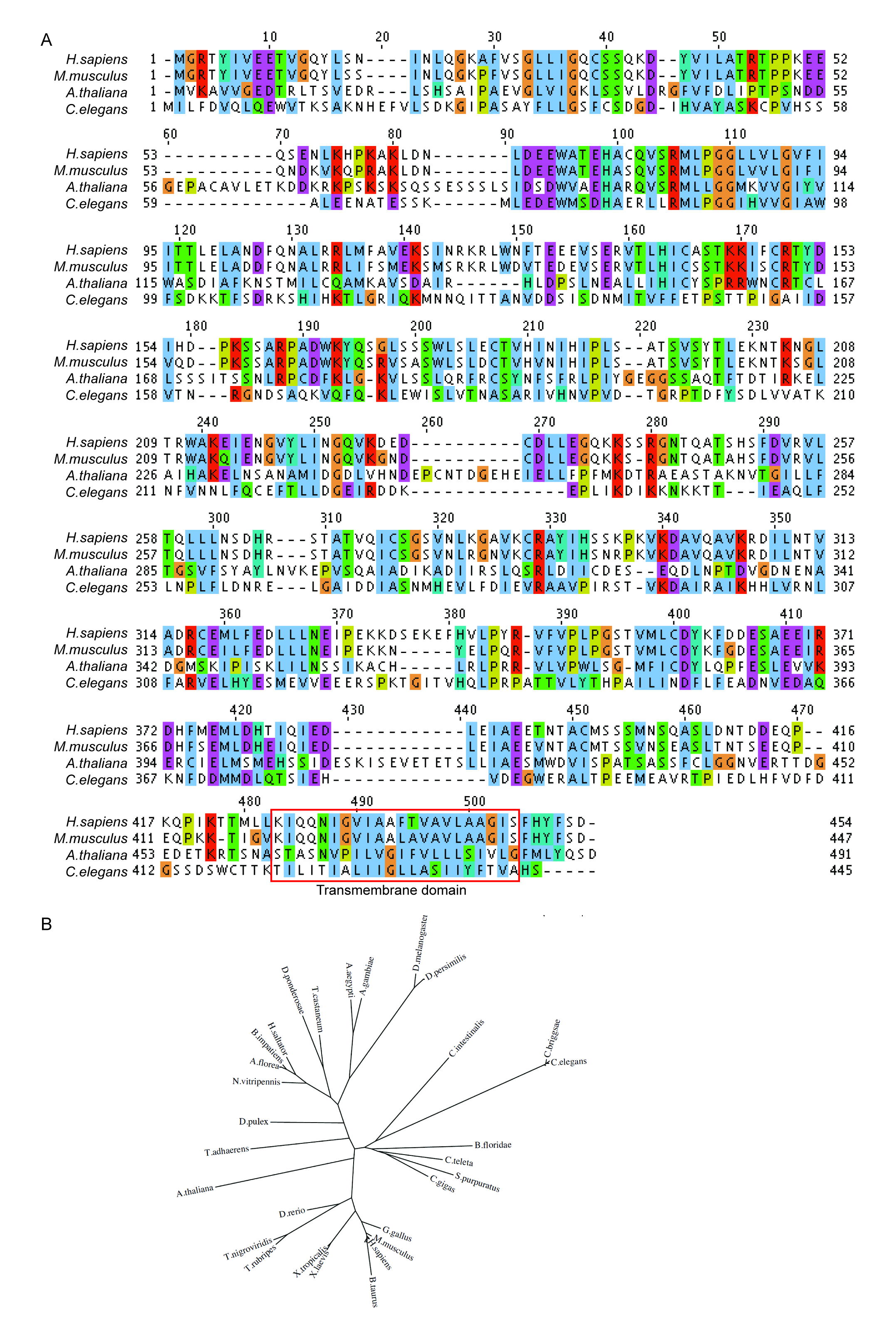

Supplement: Figure S3 — ODR-4 across phylogeny. (A) Alignment of ODR-4 from mouse, human, C. elegans and fish. Colors refer to amino acid types. (B) Phylogenetic tree of ODR-4 orthologues, showing that this protein, like UfSP2, is found in many eukaryotes. (TIF) [file pgen.1004082.s003.tif]

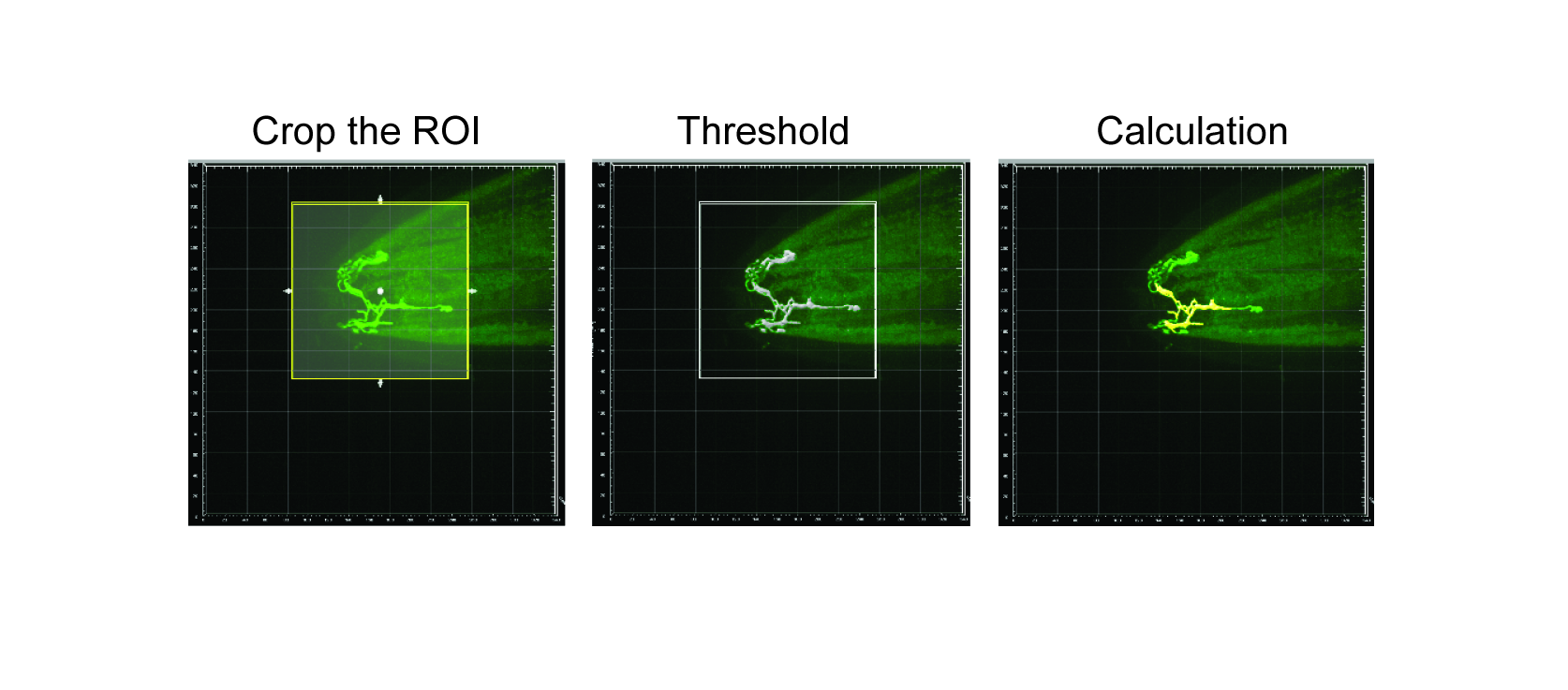

Supplement: Figure S4 — Quantification of ODR-10-GFP localization in the cilia of AWA neurons. A region of interest (ROI) was cropped and thresholded with 1200 signal intensity. Pixels with intensities over 1200 were computed and summed. (TIF) [file pgen.1004082.s004.tif]

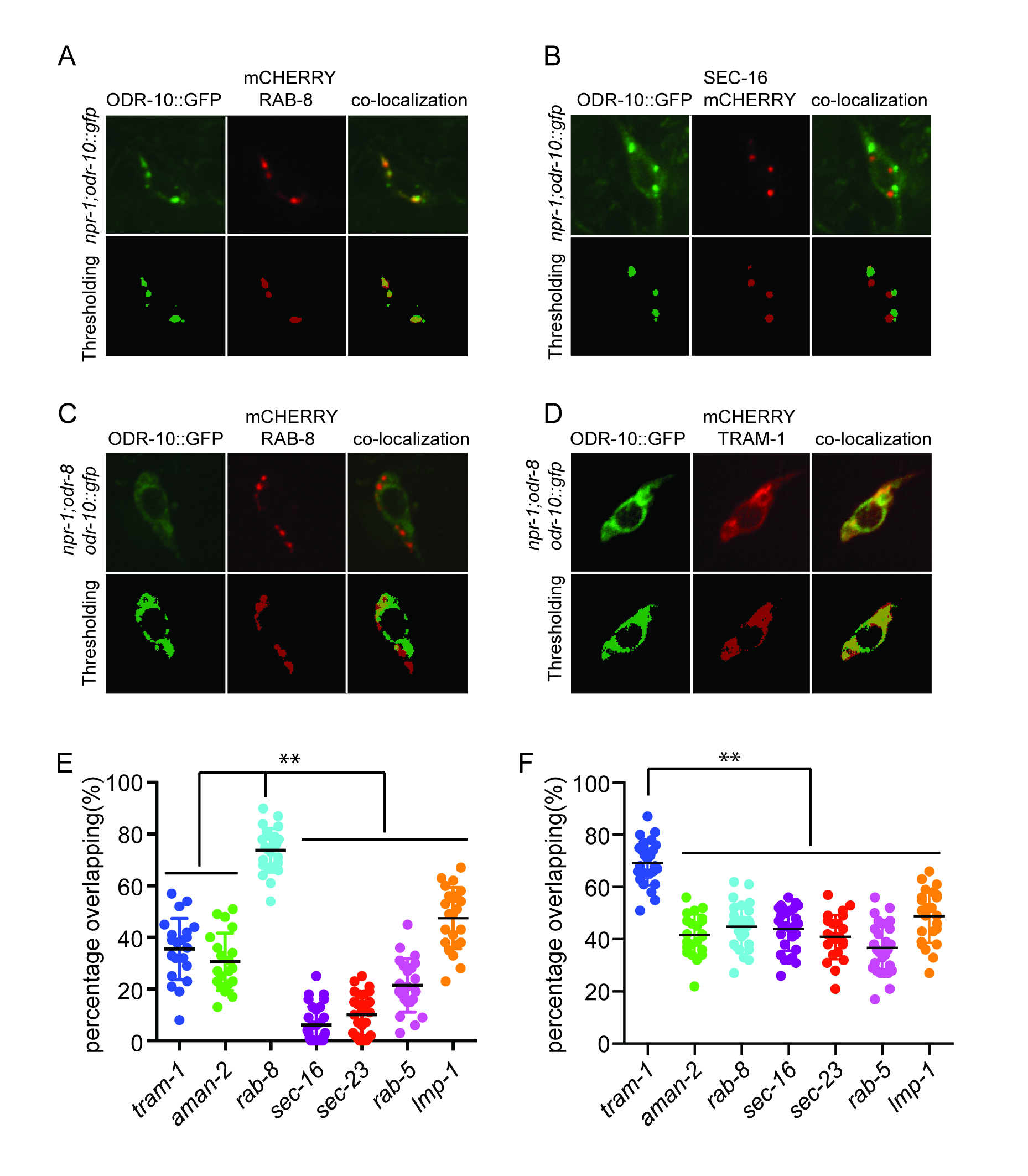

Supplement: Figure S5 — Co-localization of ODR-10-GFP with various markers in AWA neurons. (A–D) Examples of co-localization analysis of ODR-10-GFP with different markers in wild type (A–B) and odr-8 mutants (C–D). See Methods for details. (E–F). Quantification of ODR-10-GFP co-localization with different markers in wild type (E), and odr-8 mutants (F). ** indicates p<0.01 by ANOVA, Bonferroni's multiple comparisons test. (TIF) [file pgen.1004082.s005.tif]

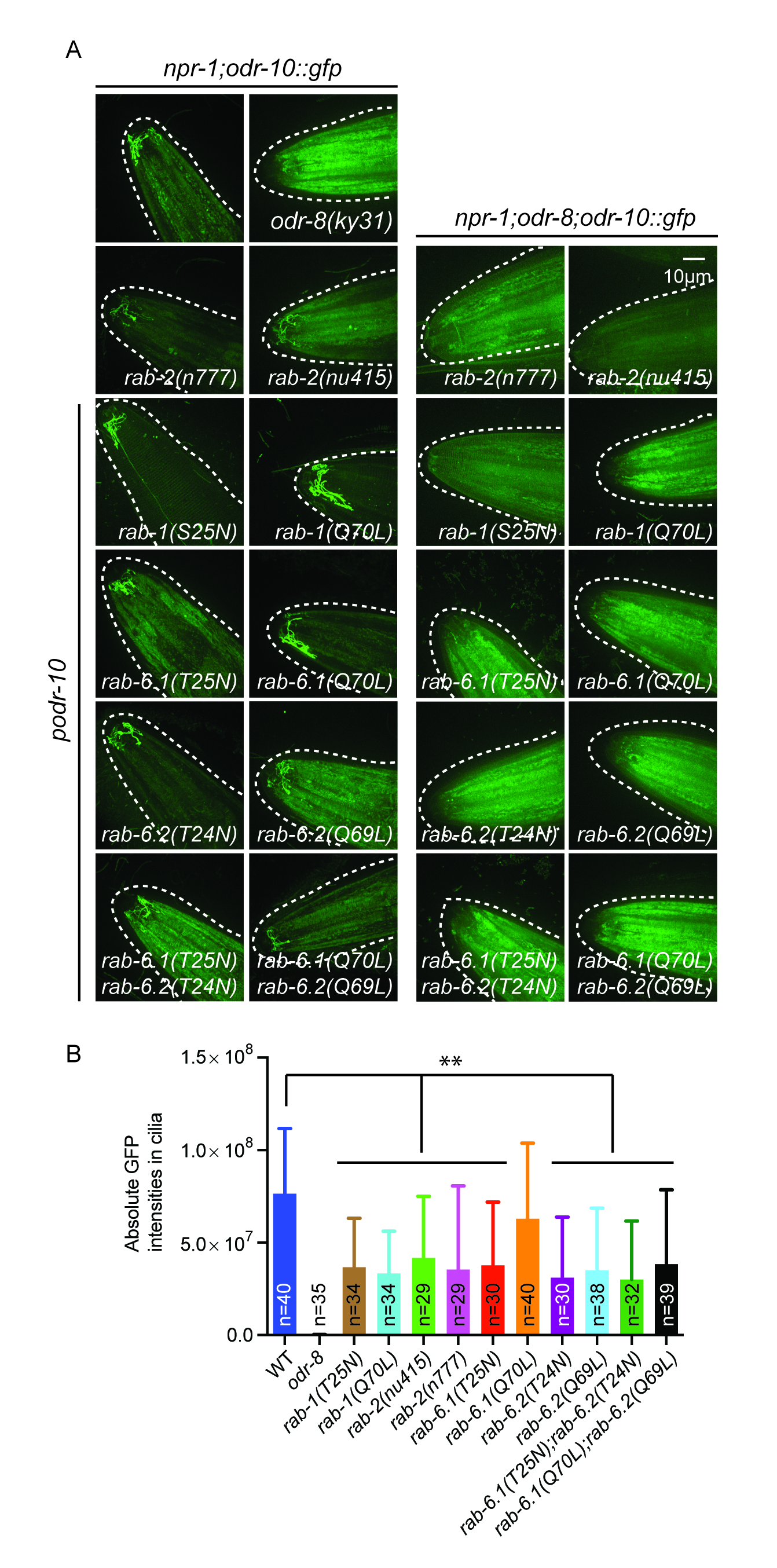

Supplement: Figure S6 — ODR-10-GFP localization in mutants expressing defective rab proteins. (A) Localization of ODR-10-GFP in animals expressing defective rab proteins in AWA neurons of npr-1 and odr-8(ky31); npr-1 animals. For rab-1, rab-6.1 and rab-6.2, the indicated dominant active and dominant negative versions of the RAB proteins were transgenically expressed in AWA from the odr-10 promoter. For rab-2, we used the null allele nu415 and the dominant active allele n777 [37] [39]. (B) Quantitation of ODR-10-GFP accumulation in cilia in different rab backgrounds. **, p<0.01 (ANOVA, Bonferroni's multiple comparisons test). (TIF) [file pgen.1004082.s006.tif]

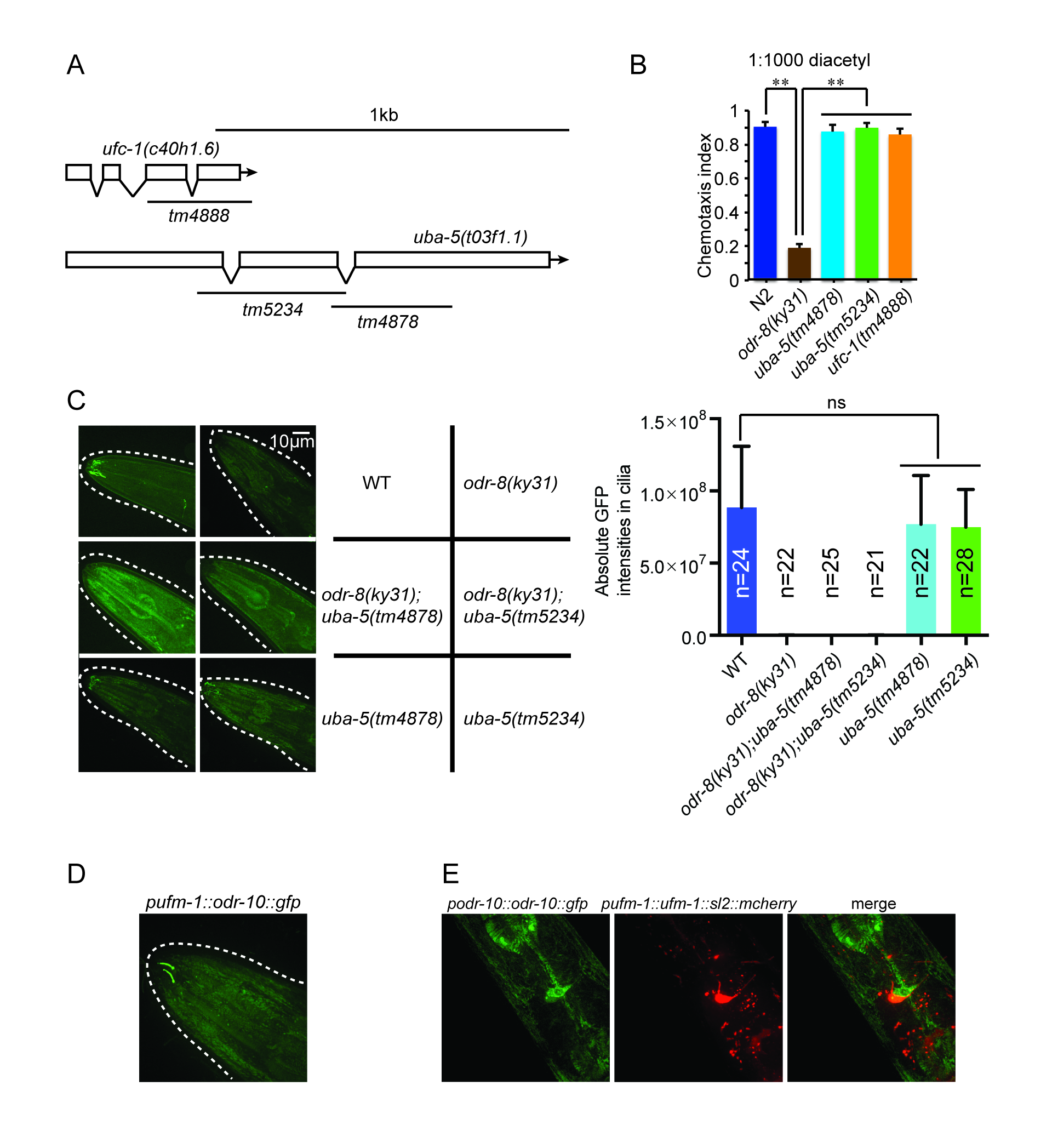

Supplement: Figure S7 — Deleting ufc-1 or uba-5 does not alter ODR-10 function. (A) Molecular nature of mutations in ufc-1 and uba-5 deletion alleles. (B) uba-5 and ufc-1 deletion mutants show wild type chemotaxis to diacetyl. (C) Deleting uba-5 does not alter the ODR-10-GFP phenotype in wild type or odr-8 mutants. (D) ODR-10::GFP expression driven by the ufm-1 promoter. (E) Expressing a ufm-1::SL2::mCherry operon from the ufm-1 promoter does not highlight AWA neurons marked with ODR-10::GFP. (TIF) [file pgen.1004082.s007.tif]

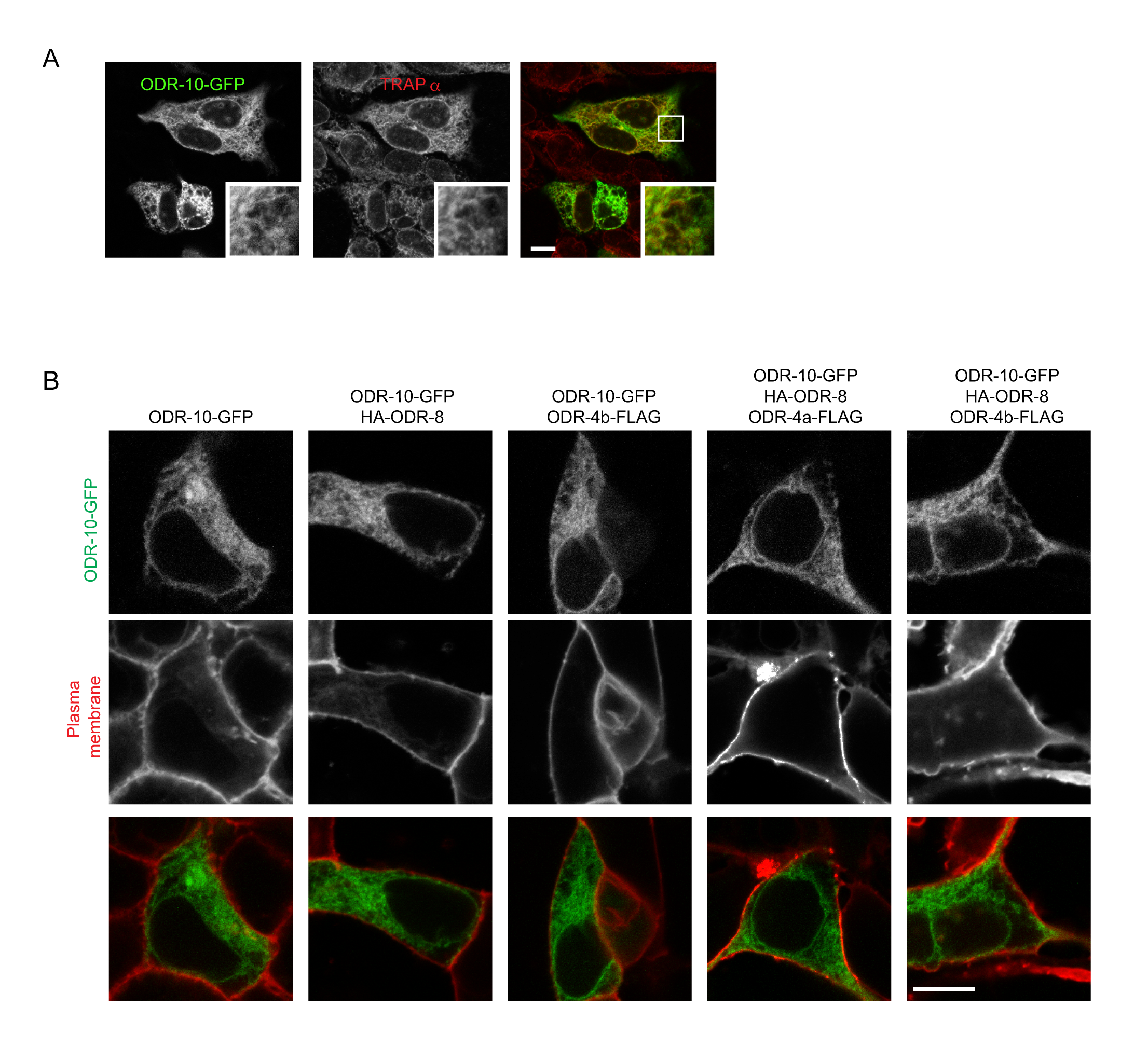

Supplement: Figure S8 — ODR-4 and ODR-8 are not sufficient to promote efficient ODR-10-GFP expression at the cell surface in HeLa cells. (A) HeLa cells were transiently transfected with ODR-10-GFP for 3 days. After fixation, the cells were subjected to immunocytochemistry by using rabbit anti- TRAPα antibodies, to highlight the ER. (B) HeLa cells were transiently transfected with indicated plasmids for 3 days. After fixation, plasma membrane was stained by PKH26. Bar, 5 µm. (TIF) [file pgen.1004082.s008.tif]
