# Supplementary material for: An ER Complex of ODR-4 and ODR-8/Ufm1 Specific Protease 2 Promotes GPCR Maturation by a Ufm1-Independent Mechanism
Source: PLoS Genet. 2014 Mar 6;10(3):e1004082. doi: 10.1371/journal.pgen.1004082 (PMC3945108; doi:10.1371/journal.pgen.1004082)
Supplement: Figure S9 — Sequence of the pEXPRESSION vector used for odr-8 mutant phenotype rescue. Green: odr-8 promoter; yellow: odr-8 genomic DNA; cyan: odr-8 3′UTR; grey: unc-54 3′UTR. (PDF) [file pgen.1004082.s009.pdf]

Sequence of the pEXPRESSION vector used for odr-8 mutant phenotype rescue:

CAGGAAACAGCTATGACCATGATTACGCCAAGCTATcaactttGTATAGAAAA
GTTGtcgcgtgaccacagttgttcataaaagattttcagcaacgaaagagaagaaaaagggacataatctgagaaataa
agctgaaaaactactgaaaataattgcgggggatttacgaaagaagaaatcgtgccatggttgcaaaatgtcgagtgcggc
gcggttttaaccgagtcacaacataaattatggagacggagacacaatgcaaacgcgcttcgctggtctttttgttttttttctt
gtaacttaaaatggaaacctggagtgatttggattcattttaattgtttttcagctttgtaagctccgctttgtacgcgattgtatga
aataaaacatgttataatttcaagttatatccaaaataaggttttcagatgtcagagtcatcgtcaaacttgaatccagatggaa
ctgagtttccagaccctaatgacgtgccaccaccatcggaagatcaattgactgaagaggtatgctgaaatattacatttgta
aaaattattaacaatctccatgttcaggatctattccggatgcgttctctgacaatcaattcgaaaaaaggacgtaagtttttttc
atcgcttgaagttgtttggaaagataattatttttaaaatatttaatattttttttagtttcagttgttggtggtgcaagtgagacagcc
gagcagtccatcatatcactcgcagattcgacttcaaattttgatgagcatgttggagatgatgccgagttggattatgagctg
gaacaagctcctgaagcaacagaagatggtgcagtggtgaacaatcctgtttgtggagggtaccatttcggaaggaaaag
agcagatgacttcgtcccaaatgccaatacaggtttcttaaaactccagttcgatgtaacactgatatctatattctataattaca
gtaacagtgctcaaatggcctacaaatctggcgattcctccacgcaaccccgtgctgagcgattctgatgaagaatggaag
aaagcgattctcgactccaccatttatctcatcggtacagctcacttttcaaaagagtcgcaggaagacgtatccaacacaatt
cgtgctgtgcaacctgattttgtcatgcttgagctgtgtccatcacgaatttcaatcatatcgatggatgaggctcggcttttga
gcgaagcgaaagatctcaactctcaaaaaattattcagaccatgaaacaagtgagttctcatatttttgttgttgtaatgttgtaa
tattttcaactttttaagaacggcgcgattcaaggaattctgcacgttcttctcctgtcaatgtcagcgcatgtaacacgtgaac
tttccatggctcccggaggagaattccgtgctgctcatcgtgcagcagttgcaactgaggtttgagtatttaaaaagttttattta
caaagtaactatttcagaattgtcgtgtagttctcggtgatcgtcctattcaagttacacttcaacgtgcactggcttctctctcca
tctggcaaaaaattcgcttcttcttccacgttgcattttcacatcgtgagaagataactgcagaagaggtatttaattattcaacc
ttttccaaaaatattccaaaattataggtggaacgatgcaagcaacgtgaccttctggagcaacttctcgccgagatggctga
tgatttcccacaactatcccaaatctttgtggaagagcgtgatgcctacatgacgcatgctttgcatatgctcgtacaccggaa
ctcagtcgaaaaacgtgcacaatggcttcgtggaacaactgggcaacaatttcaaccgttgacagttgttgctgtcgttggaa
tcggacatactccaggaattgttaacaaatggaatacaaatattgattttgatccacttatgaacatcccaccaccaagcctcg
gaacaaaggtaaattaatctgaagttattttaattctttcatttctttatttccaggttttctccactggcgtccgtattgttgtctggg
gatctttgggatatttggcttatcgtggaggacgtgctatcttcaatcgtgttagcacattcatagagtctcgttggtaggttgga
aatttttgtttaaaatttagttttgggaatgttgaaattaaacgatgaaattaaacgtgaattttgcaaaatcaaggtcgcacaga
gagaggcagcgcgtagttttgcaagcctgtggcattgttttccaccgtttttggtggtttttttcgataagttctcaattttactg
gtttcactatttttttctaatgaaaatgttacctaattcaattctcatacgattatgaggaaatttcatgttagatttgttgtaaaaacga
agtattctcacaatcttatgggaattgaattagatacataatatttttattcaaaaaaaaagaaggaaaaaccgtaccacaggg
atgagaaattacgcgaattaccaactccccattcaaagaatgaaataatagcaaacctaatctcattttcgtgcttctcccgtctt
ctctcgttattagcttttctggtatcacctgctccgcacaagttacccatgacaaccaacccactaactcgtatatcttttcagaa
caactcattgattgaagcgggcacaacagcatagcagccgtctacattcgatctacaggcgttttcatcgttattttgttctttcc
taaaatgtttatgtctaattattggacttttaatcgctttttcttgtaatgtgatacgatttctagggtgtagataggttgagaattgaa
acaaaattttcttctcccatttttttctaaactatccttttctgttaaatgcccaaacaaattgcaatttatttattagtcccacccgct
tcttccgtttatcgattgatttattctcgttttctttgtaaaatacacaaataatttaaattattcatctctctattgcaaaatccccttct
acaagtccccatattcagctcgctggtcgtataaatgacacatttcaatattattctctcgtgattcgtggtaatatccataaacta
ttattatcttttgaaaggtgattggaagagaagCAAGTTTGTACAAAAAAGCAGGCTAAGAatga
cgaattctcaaactgtctcactaattggaccaactcagatggctccacaatcaacacctccacctcctgtgaatgaattgtggt
ttattgatgcgcaggcaatgtttcaggtaatttatgtggaaatgagctatgaaagttttaagaagtgctgaaattattcgattttct
atactagttgaaaaacggaaaaaaacgaaataaaatctgaaaacactccaaaatcagtcttgcgagataaaagatttcacac
gaaatattttaagaattacgccaacctccgctcgttctccaaatccaatgccaacgaaattaatacgacaattggcggattcgt
tttcggacgtaaagctcggaaacaggttatccatgtgctattcgcctacgctgaagatctgactgaatcgaacagacaattctt
ggagagctctctgtctgctggtaattaatggaataatattattaaaaactgagatttaattcagatattgaactggttggaaactt
gaatattgatggacaatcacagattcttcctggcgggcagttcacccttcagctgacatctcgaatgcttgaaaatcgaagtat
cagcgaatttttggatatgaatgtgatgttcaacaatgaacatgttcttatggaaggagctagttgtgtcagcagagtacgttcc
cataagatctttaaattattgaataaaaacggtccgtttcaggttggatacgagtggtcacttcgagcaggtcgtgaacaaga
agatgtcaaatctgctgccgaacgtctttcaatggcttcttttcgtttcacatatctaaacgctgaacacgggttggtgatccgt

gagcagaagccagaagctgctcaacagaagtatcttgataaattctcgaaaggcgctgtaccatataaggtttggaaatact
gtctaaaattaatatgattgacggaatttcaggatgttatcgaattcactgccatgcaatctctcactagggatacatctaatgat
acggaagatcaaaagctggttccaacagttaaggttaccaagggtaaagaattttgttattttataaagatattaaattcgtaatt
tagataacaaacatttcactcggctcgtcacaattggtgaggtagttttcccagcgttctttggagattcttcactggatttgtac
aaacgatcacgtgaggcattcaatagaagagctaacaatacaatgatggttacagtcaatggaataagagctggggtaaaa
attgaaatgtaatattttttatatcgattgatatcttgcagcgcggagtaacaacgaccacatctgcaacttatcttccacctgga
tgggtttctctacttcatctccaacttccaactaaatggacggacaatgaacaaagtattcatagaaagcgtttcaacttctataa
aaattcaattttctaggaaattaccgtattcgtcttcacaagttgttcaacttgccatcatctaagccagttcttcgattgtctcaag
cgttggctctccactcagagagtgctcgtttgaccaacaagaagctcattcgagagccacatttgtctataacaaattgtatgtt
taaaaatggagtaccgaaatttgaggcattgctttgttggacccaaaactacagaatttcgtaatgagacatcaaattttgcca
acttgacagacccggaaattgcggaagtttgaatcatcaagttgtcaaaattcgagattttagctgaaaaaaaaaccgcatta
actttttcttttgagaaattttcagaacgtttcataacgaaatgcggaagtttttggcaattttgggtctaaacaagcaaagactca
gaattaattttcagatcaaccagttggtgaaataaccactgtaaatgggccgtacaattatcatcattacatgcaagatggaatt
gatgatagtggatggggatgtgcatatcgtagtttccaaactatctggagttggttcattttgaatggatatactgataaacctgt
gccaagtcatcgggaaattcagcaggttggcttaagcagatttcaattgaagttgttttatttgttgcaggcgcttgttgatattc
aagacaagcaagcgaagtttgttggttcacgacagtggattggtagtactgaaatcagttttgtgctcaatgagctactcagta
agtagttgttgagtaattgtatttaatagttaaacatttcagagttagaatgccgtttcatcgctaccaactcgggcgcagaagtt
gtcgaaagagttcgtgaacttgctcgacattttgaaacatctggaactcctgttatgatcggaggaaatatgcttgcacatact
attcttggagtcgatttcaatgataccactggagagactaagtgagtttttttttttcaaattggaagtatagcaacacaataattt
ccagattcctcgttcttgatcctcattacactggctccgaagatatcaaaacaatcacttcaaaaggatggtgtgcatggaaac
cagcgagcttctggagcaaagatcatttctacaatatggtccttcctcaaccgcctagcgatgctatttgagtgactttgtatta
ccatgcctatgaaaaacaatgcattatatatctaaattattgccaaccttctacccatttacattgtaattcctatattttttaaacct
ggattagtcctttctcgtctgtgattaatttatttcctctatagagaaatgctttctatagcccaaccaacgtttttgggaataaaa
aattggtcaaaagttgagtacaagtcaagcatggACCCAGCTTTCTTGTACAAAGTGGGTCCA
ATTACTCTTCAACATCCCTACATGCTCTTTCTCCCTGTGCTCCCACCCCCTA
TTTTTGTTATTATCAAAAAACTTCTCTTAATTTCTTTGTTTTTTAGCTTCTTT
TAAGTCACCTCTAACAATGAAATTGTGTAGATTCAAAAATAGAATTAATT
CGTAATAAAAAGTCGAAAAAAATTGTGCTCCCTCCCCCCATTAATAATAA
TTCTATCCCAAAATCTACACAATGTTCTGTGTACACTTCTTATGTTTTTTAC
TTCTGATAAATTTTTTTGAAACATCATAGAAAAAACCGCACACAAAATAC
CTTATCATATGTTACGTTTCAGTTTATGACCGCAATTTTTATTTCTTCGCAC
GTCTGGGCCTCTCATGACGTCAAATCATGCTCATCGTGAAAAAGTTTTGGA
GTATTTTTGGAATTTTTCAATCAAGTGAAAGTTTATGAAATTAATTTTCCT
GCTTTTGCTTTTTGGGGTTTCCCCTATTGTTTGTCAAGATTTCGAGGACGGC
GTTTTTCTTGCTAAAATCACAAGTATTGATGAGCACGATGCAAGAAAGAT
CGGAAGAAGGTTTGGGTTTGAGGCTCAGTGGAAGGTGAGTAGAAGTTGAT
AATTTGAAAGTGGAGTAGTGTCTATGGGGTTTTTGCCTTAAATGACAGAA
TACATTCCCAATATACCAAACATAACTGTTTCAACTTTattatacatagttgATAAT
TCACTGGCCGTCGTTTTACAACGTCGTGACTGGGAAAACCCTGGCGTTACC
CAACTTAATCGCCTTGCAGCACATCCCCCTTTCGCCAGCTGGCGTAATAGC
GAAGAGGCCCGCACCGATCGCCCTTCCCAACAGTTGCGCAGCCTGAATGG
CGAATGGCGCCTGATGCGGTATTTTCTCCTTACGCATCTGTGCGGTATTTC
ACACCGCATATGGTGCACTCTCAGTACAATCTGCTCTGATGCCGCATAGTT
AAGCCAGCCCCGACACCCGCCAACACCCGCTGACGCGCCCTGACGGGCTT
GTCTGCTCCCGGCAtccgcttacagacaagctgtgaccgtctccgggagctgcatgtgtcagaggttttcaccg
tcatcaccgaaacgcgcgagacgaaagggcctcgtgatacgcctatttttataggttaatgtcaTGATAATAATG
GTTTCTTAGACGTCAGGTGGCACTTTTCGGGGAAATGTGCGCGGAACCCC
TATTTGTTTATTTTTCTAAATACATTCAAATATGTATCCGCTCATGAGACA
ATAACCCTGATAAATGCTTCAATAATATTGAAAAAGGAAGAGTATGAGTA
TTCAACATTTCCGTGTCGCCCTTATTCCCTTTTTTGCGGCATTTTGCCTTCC

TGTTTTTGCTCACCCAGAAACGCTGGTGAAAGTAAAAGATGCTGAAGATC
AGTTGGGTGCACGAGTGGGTTACATCGAACTGGATCTCAACAGCGGTAAG
ATCCTTGAGAGTTTTCGCCCCGAAGAACGTTTTCCAATGATGAGCACTTTT
AAAGTTCTGCTATGTGGCGCGGTATTATCCCGTATTGACGCCGGGCAAGA
GCAACTCGGTCGCCGCATACACTATTCTCAGAATGACTTGGTTGAGTACTC
ACCAGTCACAGAAAAGCATCTTACGGATGGCATGACAGTAAGAGAATTAT
GCAGTGCTGCCATAACCATGAGTGATAACACTGCGGCCAACTTACTTCTG
ACAACGATCGGAGGACCGAAGGAGCTAACCGCTTTTTTGCACAACATGGG
GGATCATGTAACTCGCCTTGATCGTTGGGAACCGGAGCTGAATGAAGCCA
TACCAAACGACGAGCGTGACACCACGATGCCTGTAGCAATGGCAACAAC
GTTGCGCAAACTATTAACTGGCGAACTACTTACTCTAGCTTCCCGGCAACA
ATTAATAGACTGGATGGAGGCGGATAAAGTTGCAGGACCACTTCTGCGCT
CGGCCCTTCCGGCTGGCTGGTTTATTGCTGATAAATCTGGAGCCGGTGAGC
GTGGGTCTCGCGGTATCATTGCAGCACTGGGGCCAGATGGTAAGCCCTCC
CGTATCGTAGTTATCTACACGACGGGGAGTCAGGCAACTATGGATGAACG
AAATAGACAGATCGCTGAGATAGGTGCCTCACTGATTAAGCATTGGTAAC
TGTCAGACCAAGTTTACTCATATATACTTTAGATTGATTTAAAACTTCATT
TTTAATTTAAAAGGATCTAGGTGAAGATCCTTTTTGATAATCTCATGACCA
AAATCCCTTAACGTGAGTTTTCGTTCCACTGAGCGTCAGACCCCGTAGAA
AAGATCAAAGGATCTTCTTGAGATCCTTTTTTTCTGCGCGTAATCTGCTGC
TTGCAAACAAAAAAACCACCGCTACCAGCGGTGGTTTGTTTGCCGGATCA
AGAGCTACCAACTCTTTTTCCGAAGGTAACTGGCTTCAGCAGAGCGCAGA
TACCAAATACTGTCCTTCTAGTGTAGCCGTAGTTAGGCCACCACTTCAAGA
ACTCTGTAGCACCGCCTACATACCTCGCTCTGCTAATCCTGTTACCAGTGG
CTGCTGCCAGTGGCGATAAGTCGTGTCTTACCGGGTTGGACTCAAGACGA
TAGTTACCGGATAAGGCGCAGCGGTCGGGCTGAACGGGGGGTTCGTGCAC
ACAGCCCAGCTTGGAGCGAACGACCTACACCGAACTGAGATACCTACAGC
GTGAGCTATGAGAAAGCGCCACGCTTCCCGAAGGGAGAAAGGCGGACAG
GTATCCGGTAAGCGGCAGGGTCGGAACAGGAGAGCGCACGAGGGAGCTT
CCAGGGGGAAACGCCTGGTATCTTTATAGTCCTGTCGGGTTTCGCCACCTC
TGACTTGAGCGTCGATTTTTGTGATGCTCGTCAGGGGGGCGGAGCCTATG
GAAAAACGCCAGCAACGCGGCCTTTTTACGGTTCCTGGTCTTTTGCTGGCC
TTTTGCTCACATGTTCTTTCCTGCGTTATCCCCTGATTCTGTGGATAACCGT
ATTACCGCCTTTGAGTGAGCTGATACCAGCGGATAACAATTTCACA

Green: *podr-8*

Yellow: *odr-8* genomic DNA sequaence

Cyan: *odr-8* 3’UTR

Grey: *unc-54* 3’UTR
