# Supplementary material for: An ER Complex of ODR-4 and ODR-8/Ufm1 Specific Protease 2 Promotes GPCR Maturation by a Ufm1-Independent Mechanism
Source: PLoS Genet. 2014 Mar 6;10(3):e1004082. doi: 10.1371/journal.pgen.1004082 (PMC3945108; doi:10.1371/journal.pgen.1004082)
Supplement: Table S1 — C. elegans strains used in this study. (DOCX) [file pgen.1004082.s010.docx]

| Strain number | Genotype |
| --- | --- |
|  | N2 |
| AX204 | *npr-1(ad609)X* |
| CX2386 | *odr-8(ky31)IV* |
| CX3073 | *odr-8(ky173)IV* |
| AX433 | *npr-1(ad609)X; odr-8(ky31)IV* |
| AX3158 | *npr-1(ad609)X; odr-8(ky31)IV;odr-10::gfp X* |
| AX434 | *npr-1(ad609)X; odr-8(ky173)IV* |
| AX3159 | *npr-1(ad609)X; odr-10::gfp X* |
| CX2675 | *odr-4(n2144)III* |
| AX623 | *npr-1(ad609)X; odr-4(n2144)III* |
| AX3468 | *npr-1(ad609)X; odr-4(n2144)III; odr-10::gfp X* |
| AX3203 | *npr-1(ad609)X; odr-8(ky31)IV;odr-10::gfp X;dbEX618[podr-8::odr-8::sl2::mcherry; ccGFP]* |
| AX3504 | *npr-1(ad609)X; odr-8(ky31)IV;odr-10::gfp X;dbEX638[psrh-220::odr-8::sl2::mcherry; ccGFP]* |
| AX3194 | *npr-1(ad609)X; odr-8(ky31)IV;odr-10::gfp X;dbEX617[podr-10::odr-8::sl2::mcherry; ccGFP]* |
| AX3968 | N2;*dbEX659[podr-10::str-112::mcherry; ccGFP]* |
| AX3970 | N2;*dbEX660[podr-10::str-113::mcherry; ccGFP]* |
| AX4040 | *npr-1(ad609)X; odr-8(ky31)IV;odr-10::gfp X; dbEX660[podr-10::str-113::mcherry; ccGFP]* |
| AX4041 | *npr-1(ad609)X; odr-8(ky31)IV;odr-10::gfp X; dbEX659[podr-10::str-112::mcherry; ccGFP]* |
| AX4201 | *npr-1(ad609)X; odr-8(ky31)IV;odr-10::gfp X;dbEX704[podr-3::odr-8::sl2::mcherry; ccGFP]* |
| AX409 | *npr-1(ad609)X;odr-4(n2144)III;lin-15(n765)X;odr-4-4* |
| AX4102 | *npr-1(ad609)X; odr-8(ky31)IV;odr-10::gfp X; dbEX701[podr-10::mcherry::odr-8; ccGFP]* |
| AX4092 | *N2; dbEX698[podr-8::mcherry::odr-8; ccGFP]* |
| AX3993 | *npr-1(ad609)X;odr-10::gfp X; dbEX669[podr-10::c49c8.6::mcherry; ccGFP]* |
| AX3995 | *npr-1(ad609)X; odr-8(ky31)IV;odr-10::gfp X; dbEX669[podr-10::c49c8.6::mcherry; ccGFP]* |
| AX4063 | *npr-1(ad609)X;odr-10::gfp X; dbEX695[podr-10::mcherry::tram-1; ccGFP]* |
| AX4065 | *npr-1(ad609)X; odr-8(ky31)IV;odr-10::gfp X; dbEX695[podr-10::mcherry::tram-1; ccGFP]* |
| AX3997 | *npr-1(ad609)X;odr-10::gfp X; dbEX625[podr-10::aman-2::mcherry; ccGFP]* |
| AX3999 | *npr-1(ad609)X;odr-8(ky31)IV;odr-10::gfp X; dbEX625[podr-10::aman-2::mcherry; ccGFP]* |
| AX4027 | *npr-1(ad609)X;odr-10::gfp X; dbEX674[podr-10::mcherry::rab-8; ccGFP]* |
| AX4075 | *npr-1(ad609)X; odr-8(ky31)IV;odr-10::gfp X; dbEX674[podr-10::mcherry::rab-8; ccGFP]* |
| AX4029 | *npr-1(ad609)X;odr-10::gfp X; dbEX675[podr-10::mcherry::rab-8; ccGFP]* |
| AX4077 | *npr-1(ad609)X; odr-8(ky31)IV;odr-10::gfp X; dbEX675[podr-10::mcherry::rab-8; ccGFP]* |
| AX4031 | *npr-1(ad609)X;odr-10::gfp X; dbEX676[podr-10::sec-23::mcherry; ccGFP]* |
| AX4079 | *npr-1(ad609)X;odr-8(ky31)IV;odr-10::gfp X; dbEX676[podr-10::sec-23::mcherry; ccGFP]* |
| AX4067 | *npr-1(ad609)X; odr-8(ky31)IV;odr-10::gfp X; dbEX680[podr-10::mcherry::rab-5; ccGFP]* |
| AX4057 | *npr-1(ad609)X;odr-10::gfp X; dbEX691[podr-10::mcherry::rab-5; ccGFP]* |
| AX4069 | *npr-1(ad609)X;odr-8(ky31)IV;odr-10::gfp X; dbEX681[podr-10::lmp-1::mcherry; ccGFP]* |
| AX4059 | *npr-1(ad609)X;odr-10::gfp X; dbEX681[podr-10::lmp-1::mcherry; ccGFP]* |
| AX4104 | *npr-1(ad609)X;odr-10::gfp X;unc-101(m1)I* |
| AX4106 | *npr-1(ad609)X;odr-8(ky31)IV;odr-10::gfp X;unc-101(m1)I* |
| AX3424 | *npr-1(ad609)X; odr-8(ky31)IV;odr-10::gfp X;dbEX621[podr-8::odr-8(C421S)::sl2::mcherry; ccGFP]* |
| AX3427 | *npr-1(ad609)X; odr-8(ky31)IV;odr-10::gfp X;dbEX622[podr-8::odr-8(H547 to A)::sl2::mcherry; ccGFP]* |
| AX3484 | *npr-1(ad609)X; odr-8(ky31)IV;odr-10::gfp X;dbEX632[podr-8::odr-8(C421A)::sl2::mcherry; ccGFP]* |
| AX3197 | *uba-5(tm5234)I* |
| AX3201 | *npr-1(ad609)X;odr-10::gfp X; uba-5(tm5234)I* |
| AX3202 | *npr-1(ad609)X;odr-8(ky31)IV;odr-10::gfp X; uba-5(tm5234)I* |
| AX3196 | *uba-5(tm4878)I* |
| AX3199 | *npr-1(ad609)X;odr-10::gfp X; uba-5(tm4878)I* |
| AX3200 | *npr-1(ad609)X;odr-8(ky31)IV;odr-10::gfp X; uba-5(tm4878)I* |
| AX3198 | *ufc-1(tm4888)III* |
| VC925 | *coq-5;ufm-1(gk379)/hT2[bli-4(e937)let-?(q782)qIs48](I;III)* |
| AX4160 | *coq-5;ufm-1(gk379)/hT2[bli-4(e937)let-?(q782)qIs48](I;III);dbEX703[9.1kb DNA fragment, ufm-1(S2stop);podr-10::odr-10::gfp;ccRFP]* |
| AX4191 | *odr-8(ky31)IV; coq-5;ufm-1(gk379)/hT2[bli-4(e937)let-?(q782)qIs48](I;III);dbEX703[9.1kb DNA fragment, ufm-1(S2stop);podr-10::odr-10::gfp;ccRFP]* |
| AX4196 | *odr-8(ky31)IV; dbEX703[9.1kb DNA fragment, ufm-1(S2stop);podr-10::odr-10::gfp;ccRFP]* |
| AX4051 | *npr-1(ad609)X;odr-10::gfp X;dbEX685[podr-10::rab-1(S20N)::SL2::mcherry;ccGFP]* |
| AX4056 | *npr-1(ad609)X;odr-10::gfp X;dbEX690[podr-10::rab-1(Q70L)::SL2::mcherry;ccGFP]* |
| AX4049 | *npr-1(ad609)X;odr-10::gfp X;dbEX683[podr-10::rab-6.1(T25N)::SL2::mcherry;ccGFP]* |
| AX4053 | *npr-1(ad609)X;odr-10::gfp X;dbEX687[podr-10::rab-6.1(Q70L)::SL2::mcherry;ccGFP]* |
| AX4052 | *npr-1(ad609)X;odr-10::gfp X;dbEX686[podr-10::rab-6.2(T24N)::SL2::mcherry;ccGFP]* |
| AX4050 | *npr-1(ad609)X;odr-10::gfp X;dbEX684[podr-10::rab-6.2(Q69L)::SL2::mcherry;ccGFP]* |
| AX4054 | *npr-1(ad609)X;odr-10::gfp X;dbEX688[podr-10::rab-6.1(T25N)::SL2::mcherry; podr-10::rab-6.2(T24N)::SL2::mcherry;ccGFP]* |
| AX4048 | *npr-1(ad609)X;odr-10::gfp X;dbEX682[podr-10::rab-1(Q70L)::SL2::mcherry;podr-10::rab-6.2(Q69L)::SL2::mcherry;ccGFP]* |
| AX4081 | *npr-1(ad609)X;odr-8(ky31)IV;odr-10::gfp X;dbEX685[podr-10::rab-1(S20N)::SL2::mcherry;ccGFP]* |
| AX4082 | *npr-1(ad609)X;odr-8(ky31)IV;odr-10::gfp X;dbEX690[podr-10::rab-1(Q70L)::SL2::mcherry;ccGFP]* |
| AX4083 | *npr-1(ad609)X;odr-8(ky31)IV;odr-10::gfp X;dbEX683[podr-10::rab-6.1(T25N)::SL2::mcherry;ccGFP]* |
| AX4084 | *npr-1(ad609)X;odr-8(ky31)IV;odr-10::gfp X;dbEX687[podr-10::rab-6.1(Q70L)::SL2::mcherry;ccGFP]* |
| AX4085 | *npr-1(ad609)X;odr-8(ky31)IV;odr-10::gfp X;dbEX686[podr-10::rab-6.2(T24N)::SL2::mcherry;ccGFP]* |
| AX4086 | *npr-1(ad609)X;odr-8(ky31)IV;odr-10::gfp X;dbEX684[podr-10::rab-6.2(Q69L)::SL2::mcherry;ccGFP]* |
| AX4087 | *npr-1(ad609)X;odr-8(ky31)IV;odr-10::gfp X;dbEX688[podr-10::rab-6.1(T25N)::SL2::mcherry; podr-10::rab-6.2(T24N)::SL2::mcherry;ccGFP]* |
| AX4088 | *npr-1(ad609)X;odr-8(ky31)IV;odr-10::gfp X;dbEX682[podr-10::rab-1(Q70L)::SL2::mcherry;podr-10::rab-6.2(Q69L)::SL2::mcherry;ccGFP]* |
| AX4055 | *N2;dbEX689[pufm-1::odr-10::gfp;ccRFP]* |
| AX3985 | *npr-1(ad609)X;odr-8(ky31)IV;odr-10::gfp X;dbEX665[pufm-1::ufm-1::sl2::mcherry;ccGFP]* |
| AX4209 | *rab-2(nu415);npr-1(ad609);odr-8(ky31);odr-10::gfp* |
| AX4211 | *rab-2(n777);npr-1(ad609);odr-8(ky31);odr-10::gfp* |
| AX4262 | *rab-2(n777);npr-1(ad609);odr-10::gfp* |
| AX4264 | *rab-2(nu415);npr-1(ad609);odr-10::gfp* |
| AX4271 | *npr-1(ad609);odr-10::gfp;unc-101(m1);dbEX695[podr-10::mcherry::tram-1;ccGFP]* |
| AX4273 | *npr-1(ad609);odr-8(ky31);odr-10::gfp;unc-101(m1);dbEX695[podr-10::mcherry::tram-1;ccGFP]* |
| AX4275 | *npr-1(ad609);odr-8(ky31);odr-10::gfp;ufm-1(db1181)* |
| AX4277 | *npr-1(ad609);odr-8(ky31);odr-10::gfp;ufm-1(db1182)* |
| AX4279 | *npr-1(ad609);odr-8(ky31);odr-10::gfp;ufm-1(db1183)* |
| AX4312 | *npr-1(ad609);odr-10::gfp;ufm-1(db1181)* |
| AX4314 | *npr-1(ad609);odr-10::gfp;ufm-1(db1182)* |
| AX4316 | *npr-1(ad609);odr-10::gfp;ufm-1(db1183)* |
